# Kupffer cells dictate hepatic responses to the atherogenic dyslipidemic insult

**DOI:** 10.1038/s44161-024-00448-6

**Published:** 2024-03-11

**Authors:** Giada Di Nunzio, Sanna Hellberg, Yuyang Zhang, Osman Ahmed, Jiawen Wang, Xueming Zhang, Hanna M. Björck, Veronika Chizh, Ruby Schipper, Hanna Aulin, Roy Francis, Linn Fagerberg, Anton Gisterå, Jari Metso, Valentina Manfé, Anders Franco-Cereceda, Per Eriksson, Matti Jauhiainen, Carolina E. Hagberg, Peder S. Olofsson, Stephen G. Malin

**Affiliations:** 1 Department of Medicine Solna, Division of Cardiovascular Medicine, Center for Molecular Medicine, Karolinska Institutet, Stockholm, Sweden; 2 Xi’an Jiaotong University Health Science Center, Xi’an, China; 3 Department of Biochemistry, Faculty of Medicine, Khartoum University, Khartoum, Sudan; 4 Northeast Asia Institute of Traditional Chinese Medicine, Changchun University of Traditional Chinese Medicine, Changchun, China; 5 Science for Life Laboratory, Department of Cell and Molecular Biology (ICM), National Bioinformatics Infrastructure Sweden (NBIS), Uppsala University, Uppsala, Sweden; 6 Science for Life Laboratory, Department of Protein Science, KTH Royal Institute of Technology, Stockholm, Sweden; 7 Finnish Institute for Health and Welfare, Minerva Foundation Institute for Medical Research, Helsinki, Finland; 8 Novo Nordisk, Måløv, Denmark; 9 Section of Cardiothoracic Surgery, Department of Molecular Medicine and Surgery, Karolinska Institutet, Karolinska University Hospital, Stockholm, Sweden

**Keywords:** Dyslipidaemias, Dyslipidaemias, Innate immunity, Inflammation

## Abstract

Apolipoprotein-B (APOB)-containing lipoproteins cause atherosclerosis. Whether the vasculature is the initially responding site or if atherogenic dyslipidemia affects other organs simultaneously is unknown. Here we show that the liver responds to a dyslipidemic insult based on inducible models of familial hypercholesterolemia and APOB tracing. An acute transition to atherogenic APOB lipoprotein levels resulted in uptake by Kupffer cells and rapid accumulation of triglycerides and cholesterol in the liver. Bulk and single-cell RNA sequencing revealed a Kupffer-cell-specific transcriptional program that was not activated by a high-fat diet alone or detected in standard liver function or pathological assays, even in the presence of fulminant atherosclerosis. Depletion of Kupffer cells altered the dynamic of plasma and liver lipid concentrations, indicating that these liver macrophages help restrain and buffer atherogenic lipoproteins while simultaneously secreting atherosclerosis-modulating factors into plasma. Our results place Kupffer cells as key sentinels in organizing systemic responses to lipoproteins at the initiation of atherosclerosis.

## Abstract (web summary)

Using two complementary approaches to induce hypercholesterolemia in mice, Di Nunzio, Hellberg, Zhang, Ahmed et al. identified liver macrophages as key cells that organize the systemic responses to lipoproteins during the initial phases of atherosclerosis pathogenesis.

## Main

Apolipoprotein-B (APOB)-containing lipoproteins, including low-density lipoprotein (LDL), are the causal agents of atherosclerosis due to their ability to be retained, modified and engulfed at susceptible sites in the vasculature^1,2^. The liver is central to atherosclerotic cardovascular disease (ACVD) because it plays a primary role in APOB lipoprotein production and clearance by hepatocytes^3^. Notably, non-parenchymal liver cells, especially resident macrophage Kupffer cells (KCs), can also take up substantial amounts of APOB lipoproteins in both rats^4^ and rabbits^5^ in vivo, in human cell lines^6^ and in the specific context of hypercholesterolemic mice with hereditary hemochromatosis^7^. However, the response of the liver and its constituent cells at the initiation of atherosclerosis remains unexplored.

In addition to their well-recognized role in initiating and sustaining atherosclerosis, APOB lipoproteins can also contribute to pathologies in other organs, including the liver itself^8,9^. Metabolic dysfunction-associated steatotic liver disease (MASLD) describes a range of liver disorders that are thought to start with simple lipid accumulation—steatosis—through to irreversible cirrhosis^10^. Inquiries into how lipids contribute to MASLD have traditionally focused on free fatty acids released by dysfunctional adipose tissue. This simplified view has been challenged by the contributions of APOB lipoproteins to the disease process. MASLD and atherosclerosis also share comorbidities, including diabetes, and both can be associated with elevated levels of triglycerides and remnant APOB lipoproteins. However, MASLD confers an increased risk of ACVD beyond the sum of these individual components in a manner that is poorly understood^11,12^. Atherosclerosis and MASLD may also share similar mechanisms of disease initiation, as APOB lipoproteins can be retained in the liver by binding to heparan sulphate proteoglycans (HSPGs), similar to subendothelial retention at the onset of atherosclerosis^13,14^.

Wild-type mice have low levels of APOB lipoproteins in circulation, even when subjected to Western-style high-fat diets (HFDs), and common genetic and diet-based mouse models of MASLD often lack an elevated APOB lipoprotein component. This resultant low level of APOB lipoprotein, with cholesterol largely being transported in non-atherogenic high-density lipoprotein (HDL), excludes examining the impact of MASLD in atherosclerosis in wild-type mice. Mouse models of familial hypercholesterolemia (FH), such as the apolipoprotein E–deficient (*Apoe*^*−/−*^) and LDL receptor–deficient (*Ldlr*^*−/−*^) strains, which have been used for decades in atherosclerosis research^15^, have recently proven their utility in understanding MASLD^16^. In particular, an inflammatory role for the oxidation-specific epitopes that can form in lipoproteins has been shown in established and late-stage MASLD^9,17^.

The initiation of MASLD and, specifically, the contribution of APOB lipoproteins to the onset of primary steatosis remain largely unexplored, as the *Apoe*^*−/−*^ and *Ldlr*^*−/−*^ strains are born with elevated plasma cholesterol and, hence, in a state of allostasis. It is currently unknown which liver cells, if any, first respond to the ‘dyslipidemic insult’ caused by high concentrations of circulating atherogenic APOB lipoproteins and if such putative responses are protective or pathogenic toward ACVD. The kinetics of any such response to atherogenic dyslipidemia are also obscured: are days, weeks or months needed before a response of the liver is detected?

We hypothesized that rapidly switching the adult mouse into a state of atherogenic dyslipidemia would capture the initiation of liver steatosis mediated by APOB lipoproteins. Here we introduce two complementary approaches to achieve hypercholesterolemia, with either inducible deletion of *Apoe* or overexpression of the human PCSK9 D374Y mutation. In our strains, liver lipid accumulation was rapid, within 10 d of initiating dyslipidemia, and was accompanied by both common and strain-unique liver responses. Subjecting the mice to an HFD revealed a response dominated by genes of the immune system that was conserved between strains and correlated with human liver PCSK9 levels. By constructing a reporter mouse strain that allows for ex vivo monitoring of cellular APOB lipoprotein uptake and combining this with single-cell RNA sequencing (scRNA-seq) and deletion experiments, we reveal that KCs dominate the liver response through secretion of atherosclerosis-modulating factors but also by restraining circulating APOB lipoprotein levels. Together, we propose that understanding of atherosclerosis initiation should expand beyond vasculature-centric models.

## Results

### Mouse models for acute inducible dyslipidemia

We constructed two mouse models of inducible dyslipidemia through targeting APOE and LDLR. The rationale behind this is to discover common in vivo responses, rather than specific APOE or LDLR gene functions, after acute dyslipidemia.

**Extended Data Fig. 1:**
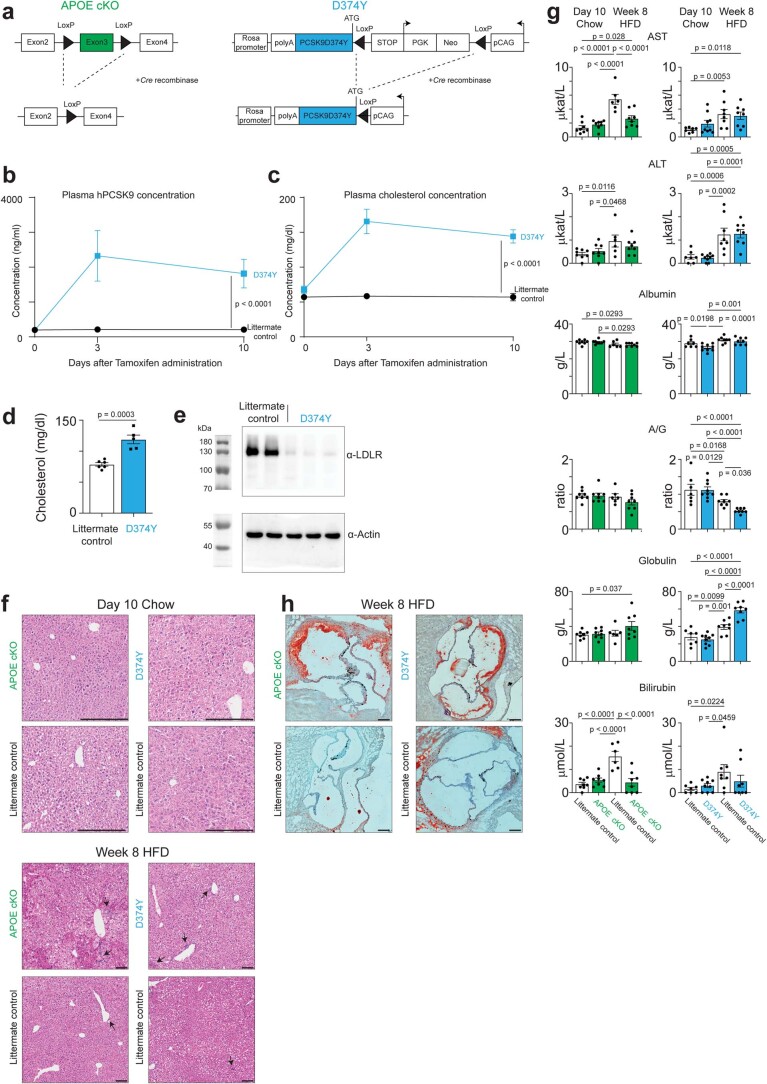
**a**, Schematic diagram of the APOE cKO and D374Y alleles. **b**, Enzyme-linked immunosorbent assay for plasma hPCSK9 protein in inducible male and female D374Y mice (blue line) and their littermate controls (black line) before and at 3 and 10 days after tamoxifen dosing (D374Y littermate control day 0: n = 1, Day 3: n = 2 and Day10: n = 5, D374Y day 0: n = 6, Day 3: n = 3 and Day10: n = 6). **c**, Plasma total cholesterol measurements (D374Y littermate control day 0: n = 6, Day 3: n = 6 and Day10: n = 6, D374Y day 0: n = 3, Day 3: n = 5 and Day10: n = 6). **d**, Cholesterol measurements 24 hours after tamoxifen administration from inducible male and female D374Y mice, (n = 6 D374Y and n = 5 littermate control mice). **e**, Immunoblot for LDLR expression in liver of D374Y mice or littermate controls after 10 days following tamoxifen induction maintained on a chow diet (n = 3 D374Y and n = 2 control, where every n represents a different mouse). **f**, Representative hematoxylin and eosin (H&E) sections of liver from APOE cKO, D374Y and respective littermate control mice at 10 days chow-fed or 8 weeks HFD diet following tamoxifen administration. Arrows indicate possible immune cell infiltration. Black scale bars represent 200 μm. n = 2 vs 2 in each group (where every n represents a different mouse) **g**, Circulating levels of AST, ALT, albumin, globulin, bilirubin and A/G ratio in dyslipidemic APOE cKO and D374Y mice with littermate controls, 10 days post tamoxifen induction while maintained on a chow diet and after 8 weeks on a high fat diet (10 days post tamoxifen: n = 8 APOE cKO and n = 8 littermate control mice; n = 9 D374Y and n = 7 littermate control mice. 8 weeks post tamoxifen while on a high fat diet: n = 8 APOE cKO and n = 6 littermate control mice; n = 8 D374Y and n = 8 littermate control mice). **h**, Histology showing ORO staining of atherosclerotic lesions in the aortic root of dyslipidemic APOE cKO and D374Y mice with littermate controls after 8 weeks on a high fat (scale bar =100 μm n = 3 vs 3 in each group (where every n represents a different mouse). All plots are mean ± SEM. Statistical analysis was performed with mixed model 2-way ANOVA (**b,c**), two-sided *t*-test (**d**) or one-way ANOVA (**g**).

To complement our previously described inducible model of dyslipidemia based upon conditional loss of *Apoe*^18^, we created a second model by inserting the human PSCK9 variant D374Y into the *ROSA26* locus. This was crossed with the tamoxifen-inducible and ubiquitously expressed *Cre* line *ROSA26*^*CreERt2*^. Hence, both conditional loss of *Apoe* (*Apoe*^*fl/−*^*ROSA26*^*CreERt2/+*^, herein referred to as APOE cKO) and inducible expression of hPCSK9 D374Y (*ROSA26*^*CreERt2/hPCSK9D374Y*^, herein referred to as D374Y) can be achieved by tamoxifen administration (Extended Data Fig. 1a). Plasma hPCSK9 increased 11-fold in D347Y mice versus littermate controls (mean, 2,325 ng ml^−1^ versus 213 ng ml^−1^) 3 d after tamoxifen (Extended Data Fig. 1b). Plasma cholesterol levels become significantly elevated already 24 h after tamoxifen dosing (Extended Data Fig. 1c,d), consistent with loss of liver LDLR protein (Extended Data Fig. 1e).

**Fig. 1:**
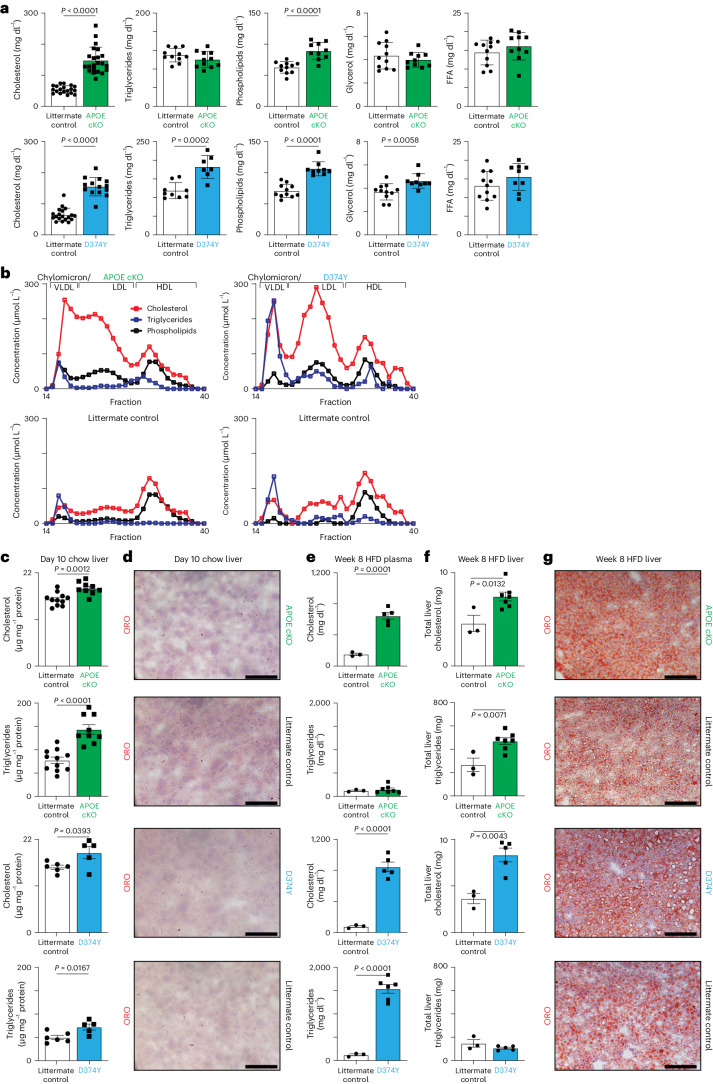
Development of mouse models for inducible APOB lipoprotein dyslipidemia. **a**, Plasma lipid levels 10 d after tamoxifen administration in APOE cKO (green bars) and D374Y (blue bars) strains together with respective littermate controls in male and female mice fed normal chow (Cholesterol: *n* = 22 APOE cKO and *n* = 19 littermate control mice; *n* = 14 D374Y and *n* = 19 littermate control mice. Triglycerides: *n* = 11 APOE cKO and *n* = 11 littermate control mice; *n* = 7 D374Y and *n* = 9 littermate control mice. PLs: *n* = 10 APOE cKO and *n* = 11 littermate control mice; *n* = 9 D374Y and *n* = 11 littermate control mice. Glycerol: *n* = 10 APOE cKO and *n* = 11 littermate control mice; *n* = 9 D374Y and *n* = 11 littermate control mice. FFA: *n* = 10 APOE cKO and *n* = 11 littermate control mice; *n* = 9 D374Y and *n* = 11 littermate control mice). **b**. Plasma lipoprotein fractionation profiles (µmol L^−1^) at 10 d after tamoxifen dosing. All curves were calculated as an average of two separately run plasma pools from male and female mice (plasma from 4–6 mice in each pool). **c**, Cholesterol and triglyceride measurements (µg mg^−1^ of protein) after liver Folch extraction from male and female mice (Cholesterol: *n* = 9 APOE cKO and *n* = 11 littermate control mice; *n* = 5 D374Y and *n* = 6 littermate control mice. Triglycerides: *n* = 9 APOE cKO and *n* = 11 littermate control mice; *n* = 5 D374Y and *n* = 6 littermate control mice). **d**, Representative pictures of liver section stained with ORO in APOE cKO and D374Y mice with respective littermate controls 10 d after dyslipidemia induction (scale bar, 100 μm). **e**, Circulating cholesterol and triglycerides (mg dl^−1^) measured in dyslipidemic APOE cKO, D374Y and respective littermate controls after 8 weeks on an HFD (Cholesterol: *n* = 5 APOE cKO and *n* = 3 littermate control mice; *n* = 5 D374Y and *n* = 3 littermate control mice. Triglycerides: *n* = 7 APOE cKO and *n* = 3 littermate control mice; *n* = 6 D374Y and *n* = 3 littermate control mice). **f**, Hepatic cholesterol and triglyceride levels measured as total liver cholesterol (mg) and total liver triglycerides (mg) in APOE cKO and D374Y with respective littermate controls (Cholesterol: *n* = 7 APOE cKO and *n* = 3 littermate control mice; *n* = 5 D374Y and *n* = 3 littermate control mice. Triglycerides: *n* = 7 APOE cKO and *n* = 3 littermate control mice; *n* = 5 D374Y and *n* = 3 littermate control mice). **g**, ORO representative liver sections of dyslipidemic APOE cKO and D374Y littermate control mice after 8 weeks on an HFD (scale bar, 100 μm). All plots are ±s.e.m. except 1a (±s.d.). For statistical analysis, a two-sided *t*-test was used (**a**,**c**,**e**,**f**). FFA, free fatty acid; VLDL, very-low-density lipoprotein. Source data

We next compared the plasma lipids in these two strains 10 d after tamoxifen administration and maintained on a chow diet (Fig. 1a). Cholesterol levels were increased in both APOE cKO (158 mg dl^−1^ versus 55 mg dl^−1^) and D374Y (150 mg dl^−1^ versus 67 mg dl^−1^) as were phospholipid levels (89 mg dl^−1^ versus 63 mg dl^−1^ in APOE cKO and 106 mg dl^−1^ versus 71 mg dl^−1^ in D374Y and controls). Noticeably, the D374Y strain displayed increased plasma levels of triglycerides (182 mg dl^−1^ versus 119 mg dl^−1^) and glycerol (4.6 mg dl^−1^ versus 3.7 mg dl^−1^), changes that were not observed in the APOE cKO mice (Fig. 1a), whereas free fatty acid levels were similar. In both the APOE cKO and D374Y strains, cholesterol was incorporated in the VLDL/chylomicron remnant fraction of lipoproteins, whereas, in D374Y, a more prominent accumulation in the LDL fraction was additionally present, as expected (Fig. 1b). The enhanced triglycerides present in D374Y mice were incorporated into the VLDL/chylomicron remnant fraction. Lipid rapidly accumulated in the liver in APOE cKO and D374Y mice fed a chow diet (Fig. 1c), but this steatosis was not immediately apparent through histological analysis (Fig. 1d and Extended Data Fig. 1f).

We next fed both strains an HFD for 8 weeks, which resulted in sustained high plasma cholesterol levels in APOE cKO and D374Y mice (mean, 646 mg dl^−1^ and 845 mg dl^−1^, respectively) and also especially high levels of triglycerides in D374Y mice (mean, 1,538 mg dl^−1^) but not in APOE cKO mice (Fig. 1e). Liver cholesterol levels were again increased in both APOE cKO and D374Y mice relative to controls, but triglyceride levels were increased only in the APOE cKO strain (Fig. 1f). This increased liver lipid content was again not manifested as clear histological differences between littermate controls and the two strains (Fig. 1g). Finally, liver function assays including aspartate aminotransferase (AST) and alanine aminotransferase (ALT) indicated impaired liver function upon switching to an HFD. However, this was not exacerbated in either the chow-fed or HFD-fed APOE cKO or D374Y strains relative to littermate controls (Extended Data Fig. 1g). Notably, after 8 weeks of HFD, fulminant atherosclerosis was present in both APOE cKO and D374Y strains, whereas the aortic roots of control mice were healthy, as expected (Extended Data Fig. 1h).

In summary, the APOE cKO and D374Y strains allow for inducible atherogenic dyslipidemia in adult mice, which results in rapid liver cholesterol and triglyceride accumulation that is not immediately apparent by histological or liver function analysis.

### A conserved inflammatory response coalesces upon sustained atherogenic dyslipidemia

**Fig. 2:**
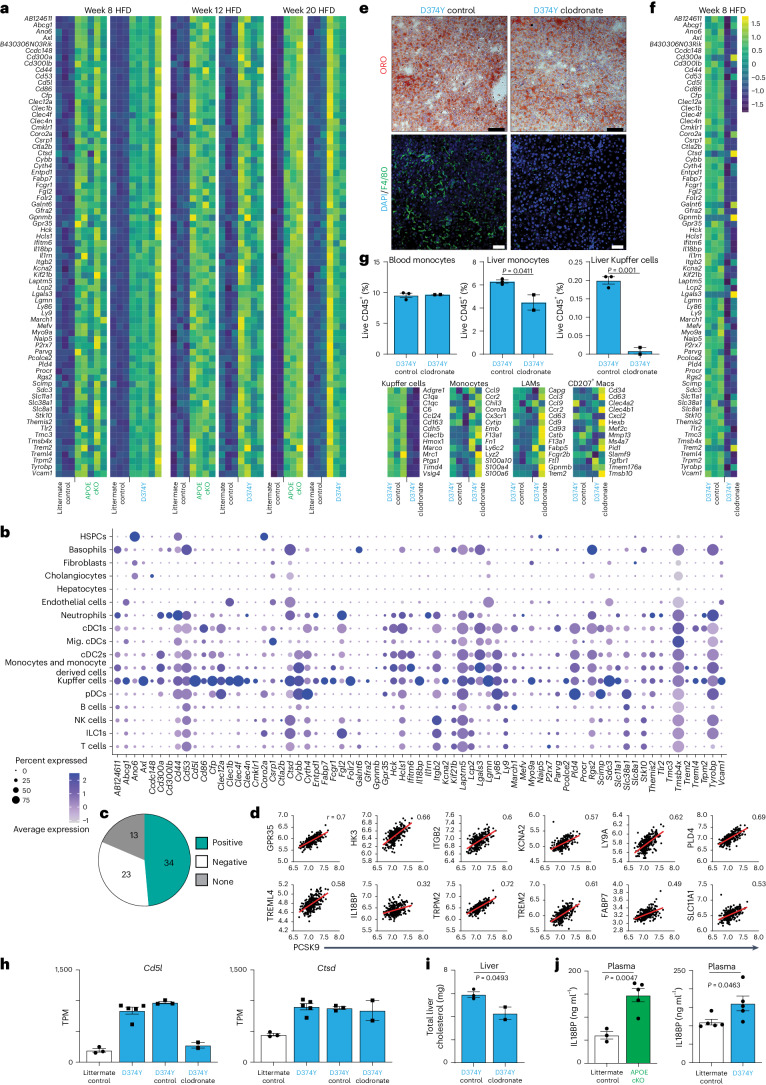
The transcriptional response of the liver to sustained dyslipidemia. **a**, Heat maps of bulk mRNA-seq of whole liver showing the 72 significant differentially expressed genes common to both dyslipidemic strains versus respective littermate controls after 8 weeks, 12 weeks and 20 weeks on an HFD (*n* = 2–5). **b**, Expression of the 72 conserved genes in liver cells using clusters taken from ref. ^19^. **c**, Pie chart showing how expression of the 72 conserved genes correlates with PCSK9 transcript levels in human liver samples (*n* = 261). **d**, Correlations with human PCSK9 levels for 12 of the 34 positively correlating genes from **c**. **e**, Representative ORO liver sections (top) and confocal images of F4/80 immunofluorescence (bottom) in the livers of D374Y dyslipidemic mice treated with clodronate liposomes and Dil liposomes (control) for 8 weeks while on an HFD (scale bar, 100 μm; *n* = 2 D3747 control and *n* = 2 D3747 clodronate, where every *n* represents a different mouse). **f**, Effect of long-term administration of clodronate liposomes on the 72 differentially expressed genes (*n* = 2–3). **g**, Top, percentages of blood and liver monocytes (live CD45^+^CD19^−^CD3e^−^CD64^−^Ly6C^+^) and liver KCs (live CD45^+^CD19^−^CD3e^−^CD64^+^Ly6C^−^F4/80^+^TIM4^+^) in D374Y dyslipidemic mice after 8 weeks of treatment with clodronate liposomes (*n* = 2 D374Y clodronate and *n* = 3 D374Y control mice). Bottom, heat maps showing variations in the expression of signature genes for KCs, monocytes, LAMs and CD207^+^ macrophages in the liver of D374Y mice after long-term clodronate exposure (*n* = 2 D374Y clodronate and *n* = 3 D374Y control mice). **h**, Transcript per million values for a clodronate-sensitive gene (Cd5l) and a clodronate-insensitive gene (Ctsd) from D374Y and littermate control and D374Y treated with Dil liposomes or clodronate liposomes (*n* = 3 littermate control, *n* = 5 D374Y, *n* = 3 D374Y control and *n* = 2 D374Y clodronate mice). **i**, Total liver cholesterol (mg) in D374Y dyslipidemic mice treated with either clodronate liposomes or Dil liposomes for 8 weeks while on an HFD (*n* = 2 D374Y clodronate and *n* = 3 D374Y control mice). **j**, Plasma levels of IL18BP (ng ml^−1^) after 8 weeks on an HFD in dyslipidemic APOE cKO and D374Y mice versus respective littermate controls (*n* = 5 APOE cKO and *n* = 3 littermate control mice; *n* = 5 D374Y and *n* = 5 littermate control mice). All plots are ±s.e.m. Two-sided *t*-test (**g**,**i**,**j**). cDC, conventional dendritic cell; HSPC, hematopoietic stem and progenitor cell; ILC, innate lymphoid cell; Macs, macrophages; Mig., migratory; NK, natural killer; pDC, plasmacytoid dendritic cell.

**Extended Data Fig. 2:**
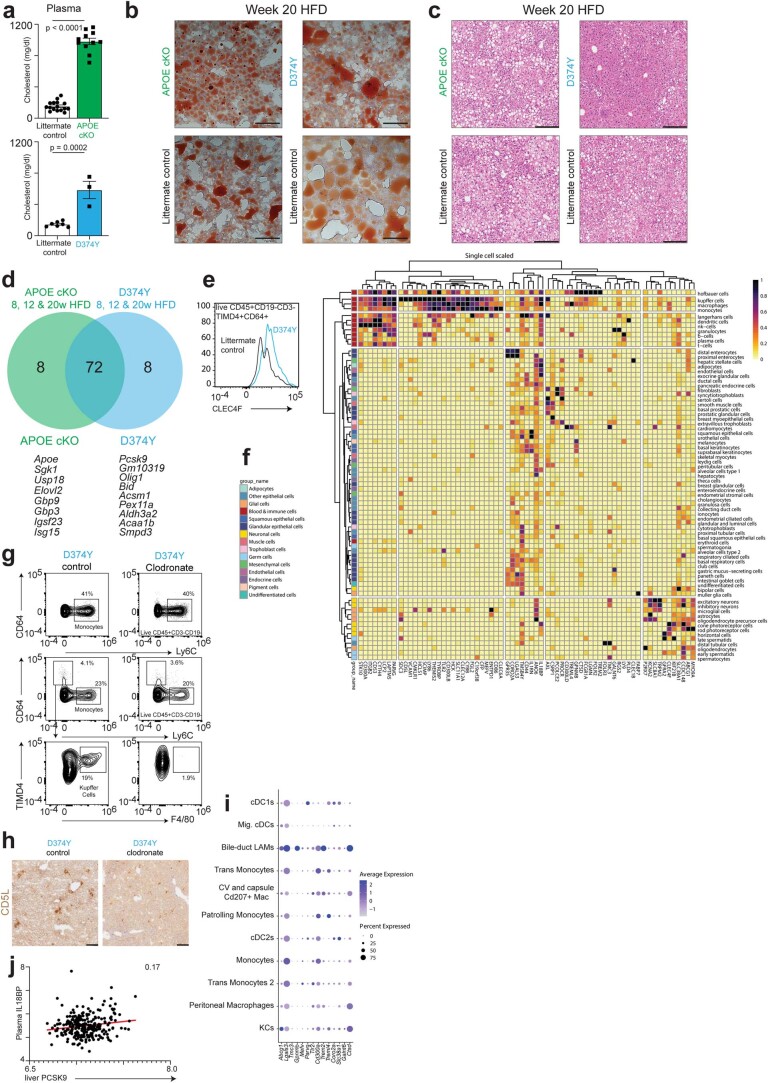
Conserved and divergent responses to sustained atherogenic dyslipidemia. **a**, Plasma levels of cholesterol in dyslipidemic APOE cKO and D374Y mice with respective littermate controls, maintained on a high fat diet for 20 weeks. (APOE cKO n = 16 vs 11; and D374Y n = 7 vs 3). Plots are ± SEM, two-sided *t*-test **b**, Representative liver sections stained with ORO in APOE cKO and D374Y mice after 20 weeks on a high fat diet (scale bars = 100 μm, n = 2 vs 2 for D374Y and n = 3 vs 3 for APOE cKO, where n indicates a different mouse). **c**, Representative liver sections stained with H&E in APOE cKO and D374Y mice after 20 weeks on a high fat diet (scale bars = 100 μm, n = 2 vs 2 in each group where n indicates a different mouse).**d**, Unique and common upregulated genes in both strains at 8, 12, and 20 weeks on a high fat diet. Unique genes for each strain are indicated at the bottom. **e**, Flow cytometry of CLEC4F levels on Kupffer cells from female D374Y versus littermate control after 8 weeks of high fat diet. **f**, Heatmap of the 72 genes upregulated at all time points in both strains, expressed in 76 human single cell types. **g**, Flow cytometry plots showing percentages of blood and liver monocytes, and liver Kupffer cells in dyslipidemic D374Y mice given clodronate liposome or Dil liposomes control for 8 weeks while on a high fat diet. **h**, Immunohistochemistry of liver to detect CD5L production in control or clodronate treated D374Y mice after 8 weeks of high fat diet (scale bars = 100 μm). **i**, The 14 genes not affected by clodronate-induced Kupffer cells depletion, expressed in liver myeloid cell clusters. **j**, Correlation between plasma IL18BP and liver *PCSK9* expression in humans.

We next sought to determine if prolonged APOB lipoprotein dyslipidemia alters the liver transcriptome. APOE cKO and D374Y strains together with littermate controls were placed on 8 weeks, 12 weeks or 20 weeks of HFD. High plasma cholesterol levels were maintained in both APOE cKO and D374Y after 20 weeks of HFD, and extensive liver lipid accumulation was present in all strains, including littermate controls (Extended Data Fig. 2a–c). Bulk mRNA sequencing (mRNA-seq) of the liver revealed 72 genes being similarly upregulated in both APOE cKO and D374Y strains compared to littermate controls at all timepoints (Fig. 2a), with only eight genes unique to either the APOE cKO or D374Y strain (Extended Data Fig. 2d). These 72 genes were strongly enriched among the resident immune cells of the liver^19^, especially KCs (identity genes *Axl*, *Clec4f*, *Cd5l* and *Folr2*) (Fig. 2b), with no hepatocyte-specific genes present. CLEC4F was also upregulated at the protein level on the cell surface of KCs (Extended Data Fig. 2e).

Next, we analyzed the 72 conserved genes in human liver samples (*n* = 261) and found that 34 of these genes also showed significant positive correlation with PCSK9 transcript levels (Fig. 2c,d) and were also enriched in human myeloid cells, including KCs, as shown by data from the Human Protein Atlas^20,21^ (Extended Data Fig. 2f). We depleted KCs using clodronate liposomes (Fig. 2e and Extended Data Fig. 2g) and performed bulk liver mRNA-seq on D374Y mice maintained on an HFD for 8 weeks. Expression of 58 of the 72 conserved genes was lost upon clodronate depletion versus liposome-control-treated D374Y mice (Fig. 2f), as was production of CD5L protein in the liver (Extended Data Fig. 2h). The 14 remaining genes not affected by KC removal were enriched in other liver myeloid populations, especially lipid-associated macrophages (LAMs) (Extended Data Fig. 2i), consistent with these surviving clodronate depletion (Fig. 2g,h). Clodronate depletion also lowered the total cholesterol content of the liver (Fig. 2i). Finally, plasma Il18BP concentration was significantly increased after 8 weeks of HFD in both strains relative to littermate controls (Fig. 2j). Il18BP plasma protein levels also positively correlated with liver PCSK9 transcription in humans (Extended Data Fig. 2j).

Altogether, we observed that the liver response to sustained atherogenic dyslipidemia is specifically characterized by a conserved myeloid and especially KC signature.

### The initial liver response to APOB lipoprotein dyslipidemia

**Fig. 3:**
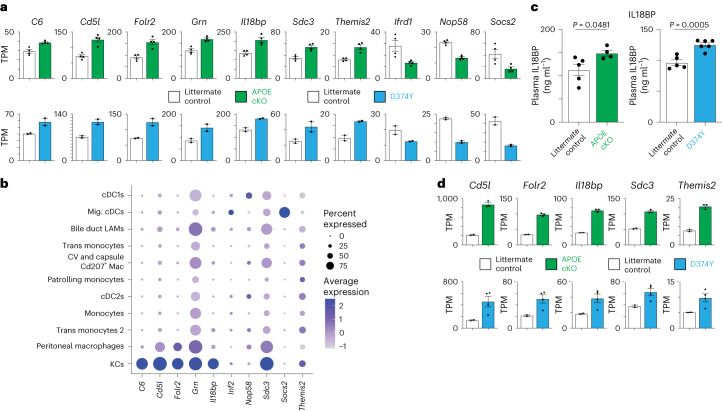
A conserved KC response at the initiation of acute APOB dyslipidemia. **a**, Transcript per million (TPM) as determined by mRNA-seq for the 10 differentially expressed genes common to both APOE cKO (*n* = 4 versus *n* = 4) and D374Y (*n* = 2 versus *n* = 2) 10 d after tamoxifen treatment. **b**, Expression of the conserved day 10 differentially expressed genes in myeloid cell clusters generated according to ref. ^19^ **c**, Secreted IL18BP (ng ml^−1^) in APOE cKO and D374Y versus respective littermate controls 10 d after tamoxifen administration (*n* = 4 APOE cKO and *n* = 5 littermate control mice; *n* = 6 D374Y and *n* = 5 littermate control mice). **d**, TPM values for five of the 10 conserved genes whose upregulation is maintained after 20 weeks on an HFD (*n* = 3 APOE cKO and *n* = 2 littermate control mice; *n* = 4 D374Y and *n* = 2 littermate control mice). All plots are ±s.e.m. Two-sided *t*-test (**c**). cDC, conventional dendritic cell; Mac, macrophage; Mig., migratory.

**Extended Data Fig. 3:**
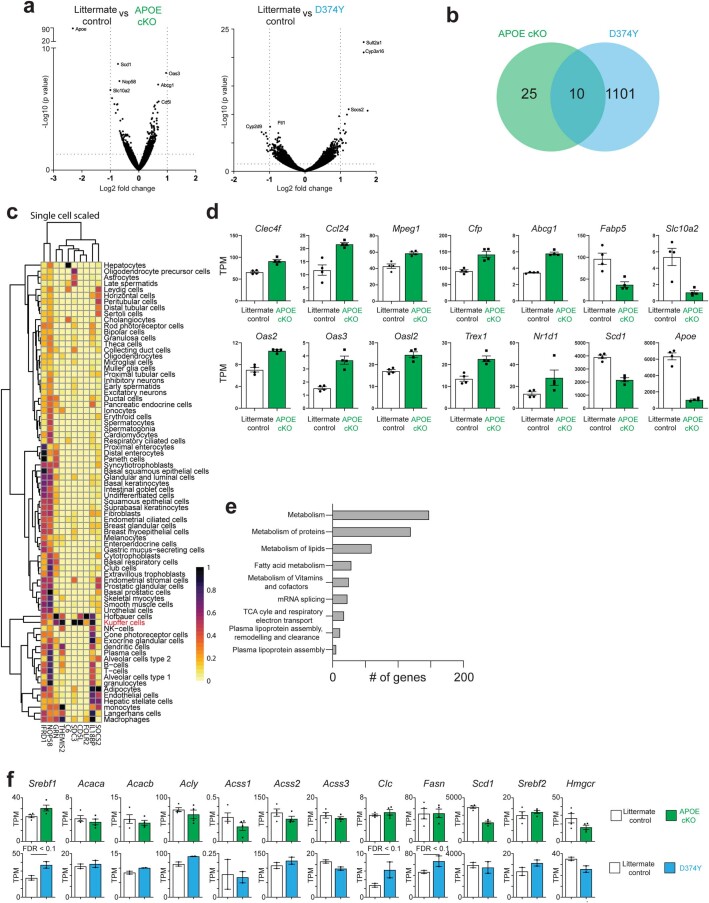
Conserved and divergent responses to initial atherogenic dyslipidemia. **a**, Volcano plots of bulk mRNA-seq of whole liver from female APOE cKO (n = 4 vs 4) and D374Y (n = 2 vs 2) mice versus their respective littermate controls (*p* value from DESeq2 two-sided Wald test). Bulk mRNA-seq was performed 10 days after tamoxifen dosing on whole liver in female mice aged 10-12 weeks at induction. **b**, Venn diagram indicating unique and overlapping differentially expressed genes. **c**, Expression in 76 human single cell types of the 10 conserved genes from APOE cKO and D374Y alleles day 10 after tamoxifen. **d**, TPM (transcript per million) of selected genes differentially regulated only in the APOE cKO strain. All plots are ± SEM and each point represents an individual liver (n = 4 vs 4). **e**, Reactome pathways of genes differentially regulated only in the D374Y strain. **f**, TPM for genes implicated in lipids synthesis pathways after 10 days post dyslipidemia induction in both strains. n = 4 APOE cKO and n = 4 littermate control; n = 2 D374Y and n = 2 littermate control. All plots are mean ± SEM. * indicates a false discovery rate < 0.1

The mouse models allow for temporal and immediate induction of atherogenic dyslipidemia. We next determined how APOB lipoprotein accumulation initially affects liver gene transcription through bulk mRNA-seq of the liver 10 d after tamoxifen with mice maintained on a normal chow diet. The APOE cKO and D374Y mice differed considerably in their initial response to acute atherogenic dyslipidemia, with 35 genes differentially expressed in the APOE cKO and 1,111 genes differentially expressed in the D374Y, as determined by bulk mRNA-seq of the whole liver (Extended Data Fig. 3a,b). However, both strains had similar dysregulation in a core set of 10 genes, including several known liver macrophage and KC identity genes (*C6*, *Cd5l*, *Folr2* and *Il18bp*) (Fig. 3a,b), and increased Il18BP protein levels could also be detected in the plasma at this early timepoint (Fig. 3c). Most of these 10 genes were also enriched in human KCs, as shown by data from the Human Protein Atlas^20,21^ (Extended Data Fig. 3c), and upregulation of five of these genes continued after 20 weeks of HFD (Fig. 3d). APOE cKO showed additional upregulation of myeloid genes (*Ccl24*, *Cfp*, *Clec4f* and *Mpeg1*), virus immunity genes (*Oas2*, *Oas3*, *Oasl2* and *Trex1*) and dysregulation of metabolic factors (*Abcg1*, *Fabp5*, *Nr1d1*, *Scd1* and *Slc10a2*), including *Apoe* itself (Extended Data Fig. 3d), whereas unique gene expression signatures in D374Y were dominated by metabolic pathways (Extended Data Fig. 3e). The steatosis observed at day 10 of dyslipidemia could not be explained by a general increase in de novo lipogenesis or cholesterol metabolism genes (Extended Data Fig. 3f). Taken together, these results indicate that the acute transition to initial atherogenic dyslipidemia in the liver is distinguished by a conserved myeloid cell response independent of lipogenesis.

### Identifying liver immune cells that recognize APOB lipoproteins

**Extended Data Fig. 4:**
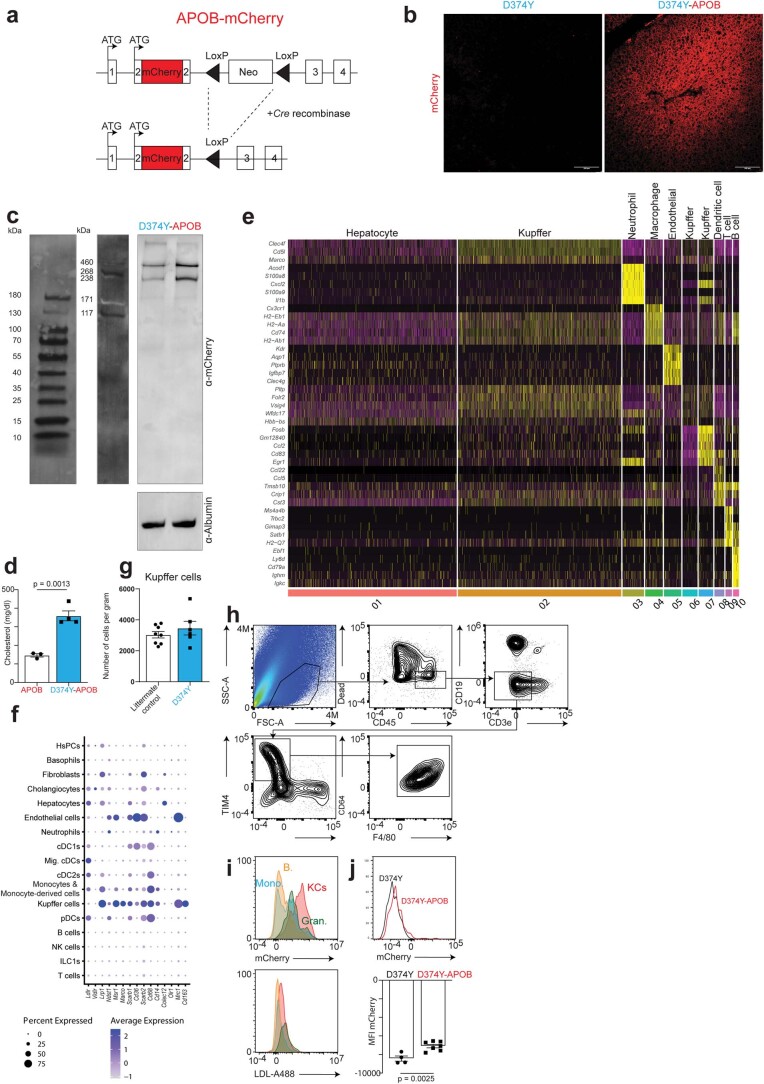
Characterisation of mCherry-APOB responsive cells. **a**, Schematic of targeting strategy of the *Apob* locus to produce mCherry-APOB. **b**, Confocal analysis of liver from D374Y and D374Y-APOB strains 10 days after tamoxifen treatment and maintained on a chow diet. Scale bars are 100μM, n = 2 D374Y-APOB and n = 4 D374Y, where n represet a different liver sections. **c**, Western blot of plasma from D374Y-APOB mice probed with anti-mCherry antibody. **d**, Plasma total cholesterol measurements from female mCherry-APOB and D374Y- mCherry-APOB after 4 weeks of high-fat diet following tamoxifen-induction (n = 4 D374Y and n = 3 littermate control mice). Plots are ± SEM. statistical analysis was performed with two-sided *t*-test. **e**, Heatmap of the top 5 marker genes for every cluster using Seurat analysis of scRNA-seq data from Live CD45+mCherry-APOB+ cells isolated from digested liver of female D374Y 10 days after tamoxifen administration and maintained on a chow diet. Yellow indicates high expression of a particular gene, and purple indicates low expression. **f**, Expression of receptors that are capable of APOB lipoproteins uptake in the cell types found in a mouse liver. **g**, Quantification of liver Kupffer cells (defined Live CD45 + CD3-CD19-Ly6G-CD64 + F4/80 + TIM4 + ) in male D374Y and littermate controls 10 days after tamoxifen administration and maintained on a chow diet. n = 6 D374Y and n = 8 littermate control mice. Data are shown as mean ± SEM. **h**, Representative gating strategy applied to Kupffer cells analysis by flow cytometry. **i**, Histogram representing the different cell populations responsible for LDL uptake in the liver of a dyslipidemic APOE cKO mouse injected i.v. with a solution containing purified LDL-AF-488. **j**, Histogram and mean fluorescent intensity for mCherry in the liver of a APOE cKO mouse injected *i.v*. with D374Y-mCherry plasma (red line), compared to a APOE cKO mouse injected with non-labelled D374Y plasma (black line) (n = 7 individual samples obtained from the liver of the injected APOE cKO mouse and n = 3 individual samples obtained from the liver of the injected littermate control mouse). Data are presented as mean ± SEM. statistical analysis was performed with two-sided *t*-test. In all cases, mice were administered tamoxifen 10 days prior and maintained on a chow diet. Source data

To characterize which cells could take up APOB lipoproteins, we tagged the APOB protein at the N-terminus with the mCherry fluorescent protein (Extended Data Fig. 4a) and crossed this together with the D374Y strain. Widespread mCherry fluorescence could be detected in the liver (Extended Data Fig. 4b), and only full-length mCherry-APOB was detected in plasma (Extended Data Fig. 4c). Cholesterol levels were noticeably increased after tamoxifen administration, indicating that mCherry-APOB was functional (Extended Data Fig. 4d).

**Fig. 4:**
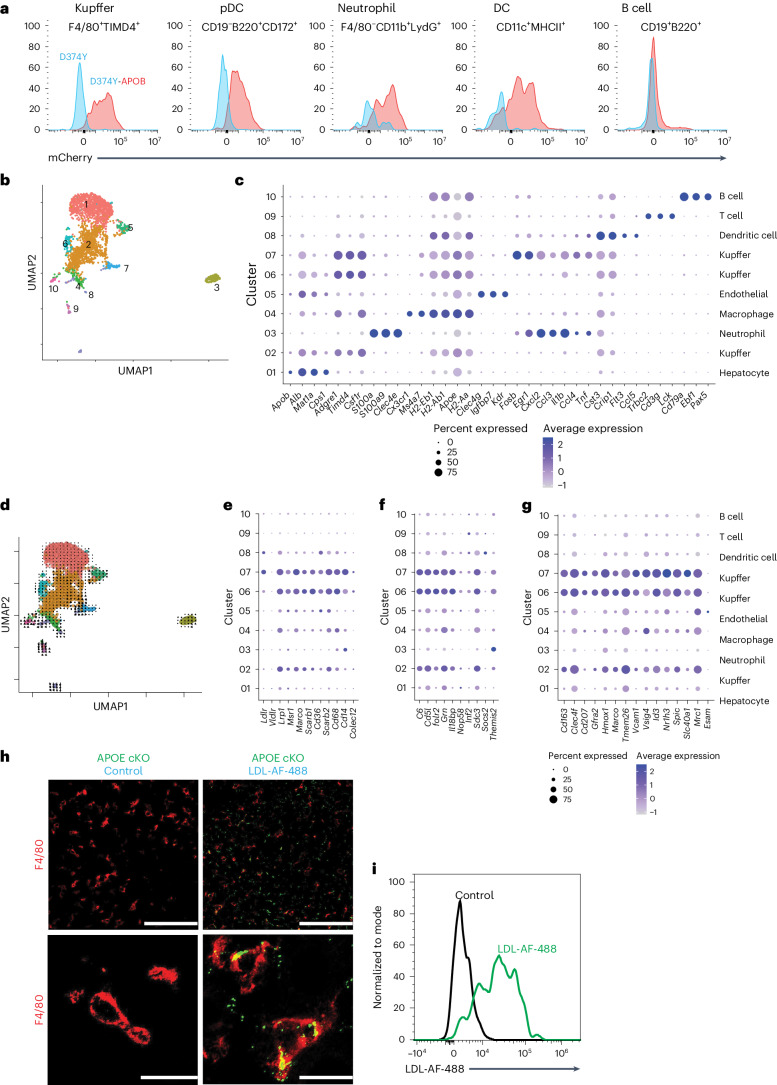
Monitoring uptake of atherogenic lipoproteins through in vivo labeling of APOB lipoproteins. **a**, Flow cytometry from immune cells extracted from the liver revealing mCherry expression in male D374Y mCherry-APOB (D374Y-APOB) or D374Y mice alone. Cells were gated as indicated above each histogram. **b**, UMAP plot indicating clusters from scRNA-seq of liver female CD45^+^mCherry^+^ cells. **c**, Dot plot for identity markers from clusters 1–10. **d**, RNA velocity analysis of the scRNA-seq data. **e**, Expression of receptors capable of APOB lipoprotein uptake in clusters 1–10. **f**, Identification of cell clusters expressing the day 10 conserved genes. **g**, Expression of core KC identity genes within each cluster. All experiments were conducted on day 10 after tamoxifen treatment, and mice were maintained on a normal chow diet. **h**, Representative confocal images of F4/80 (red) and AF-488-labeled LDL (green) in liver sections of APOE cKO mice. AF-488-labeled LDL or PBS (control) was injected intravenously into dyslipidemic APOE cKO mice that received tamoxifen 10 d prior. Mice were euthanized 30 min after injection. Scale bars, 100 μm, top panel, and 20 μm, bottom panel; *n* = 6 LDL-AF-488 and *n* = 2 control, where every *n* represents a different liver section. **i**, Flow cytometry of liver KCs showing uptake of LDL-AF-488 (LDL-AF-488 in green; control in black). DC, dendritic cell; pDC, plasmacytoid dendritic cell; UMAP, uniform manifold approximation and projection.

Analysis by flow cytometry of the liver from animals maintained on a chow diet, 10 d after tamoxifen administration, revealed multiple immune cell classes to be positive for mCherry, compared to D374Y-only mice (Fig. 4a). We sorted live CD45^+^ mCherry cells from the liver and performed 10x scRNA-seq (Fig. 4b). We identified 10 clusters (Fig. 4c and Extended Data Fig. 4e) of cells consisting of cluster 1 (hepatocytes: *Alb*, *Mat1a* and *CPs1*); clusters 2, 6 and 7 (KCs: *Adgre1, Timd4* and *Csf1r*); cluster 3 (neutrophils: *S100A8*, *S100A9* and *Clec4e*); cluster 4 (macrophages: *Cxc3r1*, *MHCII* and *Apoe*); cluster 5 (endothelial cells: *Clec4g*, *Igfbp7* and *Kdr*); cluster 8 (dendritic cells: *Cst3*, *Crip1*, *Flt3* and *MHC II*); cluster 9 (T cells: *Trbc2*, *Cd3g* and *Lck*); and cluster 10 (B cells: *Cd79a*, *Ebf1* and *Pax5*). Notably, the KC cluster 7 population additionally expressed an array of acute inflammatory genes (*Il1b*, *Tnf*, *Cxcl12*, *Ccl3*, *Ccl4*, *Fosb* and *Egr1*). RNA velocity^22^ analysis of the scRNA-seq data indicates that this subset 7 could be derived from the main KC population cluster 2 (Fig. 4d).

The three KC populations were enriched for receptors that uptake APOB lipoproteins (Fig. 4e and Extended Data Fig. 4f) and the conserved set of 10 genes dysregulated at day 10 after tamoxifen, in both APOE cKO and D374Y strains (Fig. 4f). Furthermore, the KCs were positive for the recently identified core set of KC genes (*Cd163*, *Clec4f*, *Cd207*, *Gfra2*, *Hmox1*, *Marco*, *Slc40a1*, *Tmem26*, *Vcam1* and *Vsig4*)^19,23^, including those that control lipid handling (*Id3*, *Nr1h3* and *Spic*)^24,25^, as well *Mrc1* but not *Esam* (Fig. 4g). The absolute number of KCs did not change in the liver (Extended Data Fig. 4g). We next purified and fluorescently labeled LDL from D374Y mice and injected this into APOE cKO mice. Thirty minutes after injection, LDL could be observed co-localized with F4/80^+^ cells in the liver (Fig. 4h), which was further confirmed as KCs by flow cytometry (Fig. 4i and Extended Data Fig. 4h,i). Additionally, plasma from mCherry-APOB D374Y also associated with KCs after injection into APOE cKO (Extended Data Fig. 4j).

These observations led us to conclude that, upon transition to a steatotic liver, KCs can take up APOB lipoproteins and induce an inflammatory gene expression profile.

### Liver KCs coordinate the liver response to APOB lipoprotein dyslipidemia

**Fig. 5:**
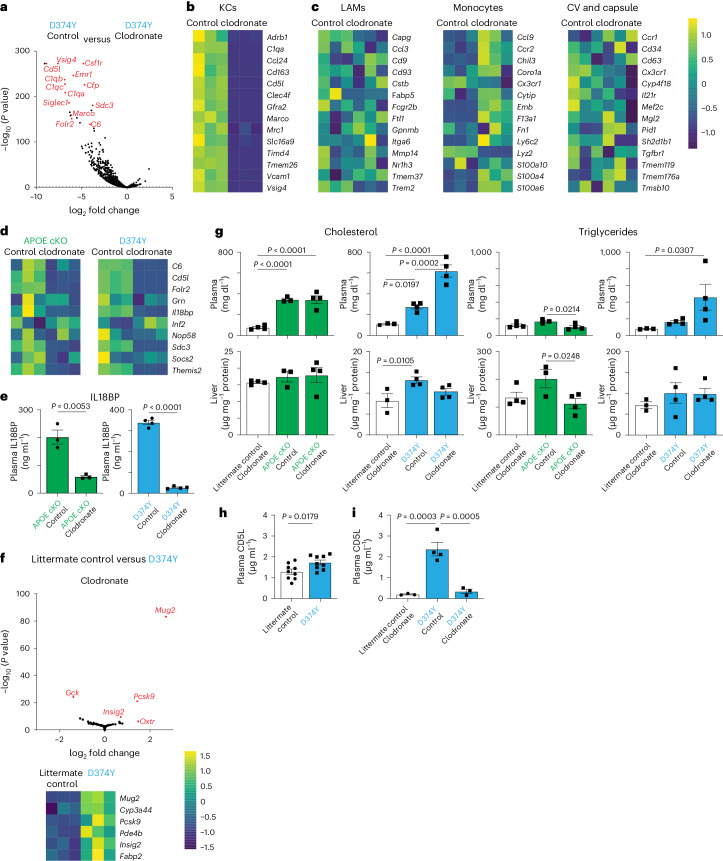
Ablating KCs prevents the hepatic response to atherogenic dyslipidemia. **a**, Volcano plot of genes downregulated in female D374Y mice treated with clodronate liposome during 10-d post-tamoxifen administration while maintained on a chow diet (*n* = 3 versus *n* = 3; *P* value from DESeq2 two-sided Wald test). **b**,**c**, Effects of clodronate liposomes on the expression of core identity genes of KCs (**b**), LAMs, monocytes and CV and capsular macrophages (**c**) in the liver of D374Y mice. **d**, Effect of clodronate liposomes on the day 10 conserved gene expression in both APOE cKO and D374Y mice. **e**, IL18BP plasma levels in dyslipidemic APOE cKO and D374Y mice given clodronate liposomes or Dil liposomes controls (ng ml^−1^, APOE cKO *n* = 3 and D374Y *n* = 4). **f**, Volcano plot and heat map indicating minimal response of the liver to dyslipidemia when comparing littermate control versus D374Y with both treated with clodronate liposomes, as determined by mRNA-seq (*n* = 3 versus *n* = 3; *P* value from DESeq2 two-sided Wald test). **g**, Total plasma (mg dl^−1^) and liver (µg mg^−1^ of protein) cholesterol and triglyceride measurements in dyslipidemic APOE cKO and D374Y mice given clodronate liposomes or Dil liposomes controls with respective littermate controls also administered clodronate liposomes (APOE cKO *n* = 4 versus *n* = 3 versus *n* = 4 and D374Y *n* = 3 versus *n* = 4 versus *n* = 3). **h**, Plasma CD5l (µg ml^−1^) concentrations as determined by ELISA in D374Y and littermate control mice 10 d after tamoxifen administration (*n* = 9 versus *n* = 9). **i**, Plasma CD5l (µg ml^−1^) concentrations in dyslipidemic D374Y mice and littermate control mice given clodronate liposomes and dyslipidemic D374Y mice administered Dil liposomes controls (*n* = 3 versus *n* = 4 versus *n* = 3). All experiments were conducted on day 10 after tamoxifen treatment, and mice were maintained on a normal chow diet. All plots are ±s.e.m. Two-sided *t*-test (**e**,**h**) or one-way ANOVA (**g**,**i**).

**Extended Data Fig. 5:**
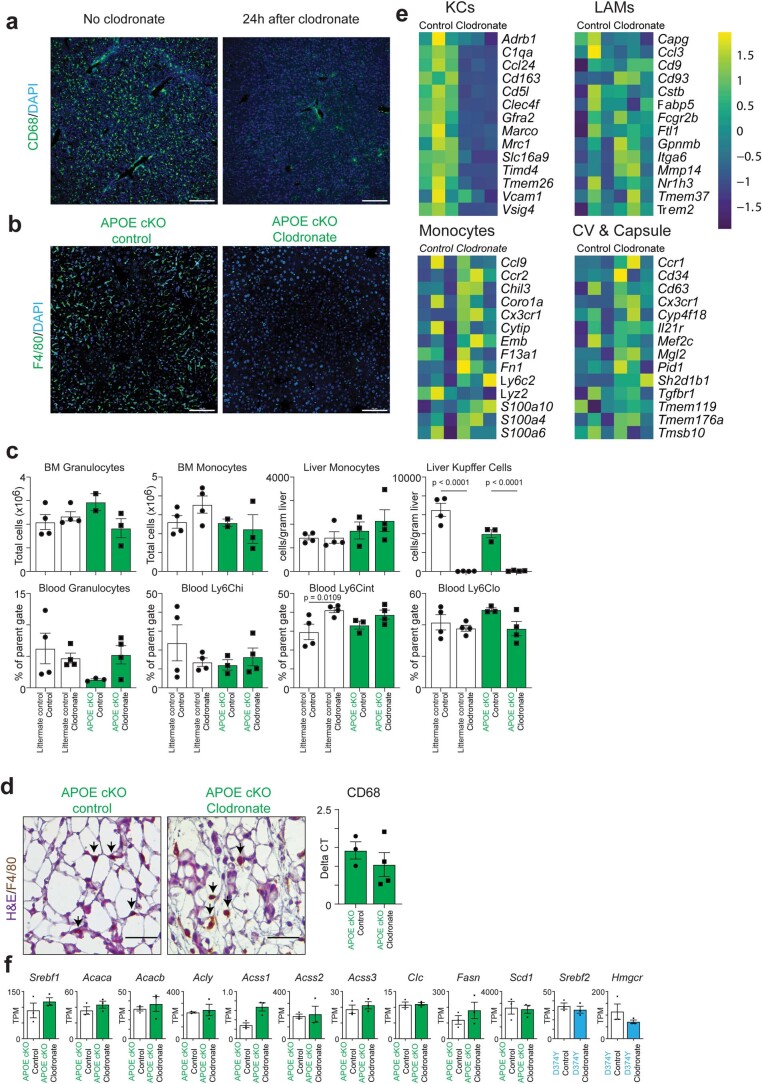
Specificity of Liver KC ablation by clodronate liposomes. **a**, Confocal microscopy of liver D374Y mice stained with anti-CD68 and b, APOE cKO mice stained with F4/80, with and without clodronate liposome treatment as indicated (scale bar =100 μm, n = 2 vs 2 in each group, where n represents a different mouse). **c**, Absolute numbers of myeloid cell types in blood, bone marrow (BM), and liver in male APOE cKO mice and littermate controls with and without clodronate treatment, as determined by Flow cytometry. Bone marrow granulocyte were defined as live CD45 + CD11b + Ly6G + , bone marrow monocytes as live CD45 + CD11b + Ly6G-Ly6C + , liver monocytes as live CD45 + CD19-CD3e-TIMD4-Ly6C+ liver Kupffer cells as CD45 + CD19-CD3e-TIMD4 + Ly6C-F4/80 + CD64 + , blood granulocytes as live CD45 + CD19-B220-CD11b + Ly6G+ and blood monocytes as live CD45 + CD19-B220-CD11b + Ly6G-Ly6Clo to hi. n = 4 littermate control (Control), n = 4 littermate control (Clodronate), n = 3 APOE cKO (Control), and n = 4 APOE cKO (Clodronate) mice. **d**, Immunohistochemical detection of F4/80 in the adipose tissue of dyslipidemic APOE cKO mice treated with clodronate or Dil liposome control (scale bar indicates 40 mm). On the right, qPCR analysis showing no difference in the expression of CD68 in the visceral white adipose tissue of the same mice. Data is presented as average delta CT normalized to TBP + /- SEM (n = 4 APOE cKO clodronate and n = 3 APOE cKO control). **e**, Effects of clodronate liposomes on Kupffer cells, LAMs, Monocytes and CV and capsular macrophages as determined by bulk RNA seq of whole liver of APOE cKO mice (n = 3 vs 3) **f**, TPM after clodronate administration showing no significant reduction in the expression of de novo lipogenesis genes in APOE cKO mice, and cholesterol synthesis genes in D374Y mice (APOE cKO n = 3 vs 3; and D374Y n = 3 vs 3). Statistical analysis was performed with one-way ANOVA (c) or two-sided *t*-test (d). All experiments were performed 10 days after tamoxifen treatment in mice maintained on a normal chow diet. All plots are ± SEM.

To determine the role of KCs during the transition to steatosis, we injected mice on a chow diet with clodronate liposomes^26^. This effectively depleted KCs (Extended Data Fig. 5a,b) but not liver monocytes (Extended Data Fig. 5c), monocytes and granulocytes in the blood or bone marrow (Extended Data Fig. 5c) or adipose tissue macrophages (Extended Data Fig. 5d). As expected, livers from the APOE cKO and D374Y strain administered with clodronate liposome, versus mice injected with control Dil liposomes, had strongly downregulated expressions of KC identity genes, such as *Clec4f*, *Timd4* and *Cd5l* (Fig. 5a,b), as determined by mRNA-seq. The molecular signature for liver monocytes, LAMs and central vein (CV) and capsule macrophages remained intact in clodronate-treated APOE cKO (Extended Data Fig. 5e) and D374Y (Fig. 5c) strains. The conserved and upregulated day 10 genes (*C6*, *Cd5l*, *Folr2*, *Grn*, *Inf2* and *Il18bp*) displayed decreased expression in clodronate-treated mice, consistent with these being KC genes (Fig. 5d). Il18BP was also significantly decreased in the plasma of clodronate-treated versus control-treated mice of both strains (Fig. 5e). We also compared littermate control mice and D374Y mice, both treated with clodronate liposomes. Strikingly, the large-scale gene expression changes normally seen at day 10 in D374Y mice were absent, with only 29 largely hepatocyte-specific genes being differentially expressed (Fig. 5f).

To exclude the possibility that KC ablation prevented atherogenic dyslipidemia as an alternative explanation for this lack of liver response, we measured plasma and liver lipids in both APOE cKO and D374 strains. Loss of KCs strongly and specifically increased the plasma concentrations of both cholesterol (approximately 600 mg dl^−1^) and triglycerides (approximately 400 mg dl^−1^) in D374Y compared to controls, even though the mice were maintained on a normal chow diet (Fig. 5g). Clodronate treatment also decreased liver cholesterol levels in D374Y mice and liver triglyceride levels in APOE cKO mice (Fig. 5g), without affecting the expression of the respective lipogenesis genes (Extended Data Fig. 5f).

CD5L is a secreted pro-atherosclerotic factor^27^ and canonical KC marker^25^. *Cd5l* was the only factor upregulated in both strains at all timepoints tested: day 10 chow diet and week 4, 8, 12 and 20 HFD. Plasma CD5L was increased in D374Y mice already at 10 d after tamoxifen on a chow diet (Fig. 5h). Furthermore, a 10-fold increase in CD5L in Dil liposome-control-treated D374Y mice versus clodronate-treated littermate controls was evident, and clodronate treatment of D374Y mice reversed this increase (Fig. 5i).

In summary, KCs are essential for the liver response to dyslipidemia and modulate plasma and liver atherogenic lipoprotein concentrations.

### Liver responses are unique to APOB lipoproteins and require CD8 T cells

**Fig. 6:**
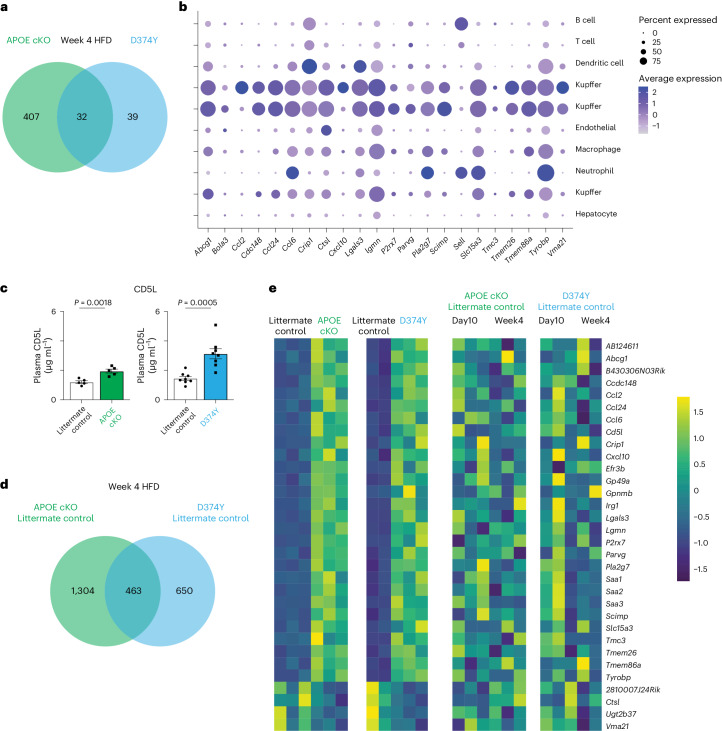
APOB lipoproteins are required for the KC inflammatory response to an HFD. **a**, Venn diagram of unique and overlapping differentially expressed liver genes after 4 weeks of HFD after tamoxifen induction in female APOE cKO (*n* = 3) and D374Y (*n* = 2 versus *n* = 3) mice versus respective littermate controls, as determined by bulk mRNA-seq. **b**, Expression of conserved genes from the week 4 HFD analysis in the liver day 10 scRNA-seq dataset. **c**, ELISA for secreted CD5L in plasma after 4 weeks on an HFD (APOE cKO *n* = 5 versus *n* = 5 and D374Y *n* = 8 versus *n* = 8). **d**, Identification of conserved transcriptional response to 4 weeks of HFD from littermate controls only of both strains. **e**, Heat map for expression of conserved genes from APOE cKO and D374Y mice compared to littermate controls alone in response to 4 weeks of HFD. All plots are ±s.e.m. Two-sided *t*-test (**c**).

**Extended Data Fig. 6:**
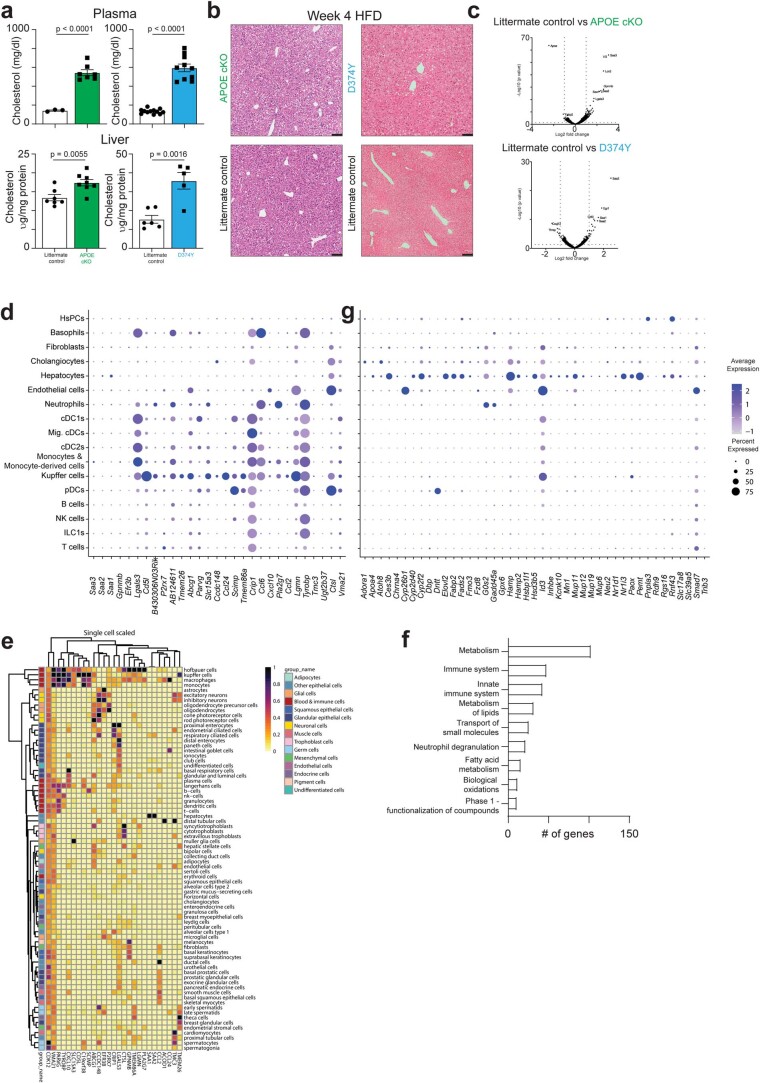
The liver response to four weeks of high-fat diet. **a**, Plasma (mg/dl) and liver (ug/mg protein) cholesterol measurements in male and female APOE cKO and female D374Y mice together with their respective littermate controls 4 weeks after Tamoxifen administration and maintained on a HFD. Plasma: n = 7 APOE cKO and n = 3 littermate control mice; n = 10 D374Y and n = 12 littermate control mice. Liver: n = 8 APOE cKO and n = 7 littermate control mice; n = 5 D374Y and n = 6 littermate control mice. All plots are ± SEM. Statistical analysis was performed with two-sided *t*-test. **b** Hematoxylin and eosin staining of paraffin sections of the liver from D374Y and littermate control mice 4 weeks after Tamoxifen administration and maintained on a HFD (scale bar =100 μm, n = 2 vs 2 in each group, where n represents a different mouse). **c**, Volcano plot of differentially expressed genes after 4 weeks of high-fat diet following tamoxifen-induction in APOE cKO or D374Y mice versus respective littermate controls as determined by mRNA-seq (*p* value from DESeq2 two-sided Wald test). **d**, Expression in liver cells of the differentially expressed genes conserved in both strains. **e**, Expression in 76 human single cell types of the 27 conserved genes from APOE cKO and D374Y alleles 4 weeks after Tamoxifen administration and maintained on a HFD. **f**, Reactome pathways of genes differentially regulated only in littermate control mice after 4 weeks of HFD. **g**, expression pattern of HFD-only induced genes in the liver cells.

To examine the role of KCs and APOB lipoproteins during the establishment of liver steatosis, we subjected our mouse strains to a short 4-week HFD regime after tamoxifen administration. This diet and timepoint further elevated cholesterol compared to day 10 on chow (Extended Data Fig. 6a), and steatosis and ballooning were clearly visible in both genotypes (Extended Data Fig. 6b). Bulk mRNA-seq determined that 28 genes were upregulated and four were downregulated in both APOE cKO and D374Y strains compared to their respective littermate controls (Fig. 6a and Extended Data Fig. 6c). Noticeably, those increased transcripts included well-established pro-inflammatory mediators, such as *Ccl2*, *Ccl6*, *Ccl24*, *Cd5l*, *Irg1*, *Lgals3*, *Lgmn*, *P2rx7, Pla2g7*, *Scimp*, *Sell*, *Slc15a3* and *Tyrobp*, and acute phase proteins *Saa1*, *Saa2* and *Saa3*. Analysis of the day 10 scRNA-seq data (Fig. 6b) and liver cell atlas (Extended Data Fig. 6d) revealed strong enrichment among the KC clusters for this inflammatory 4-week HFD signature, and the Human Protein Atlas confirmed that many of these genes were also enriched in human KCs (Extended Data Fig. 6e). Circulating CD5L also increased in plasma of both week 4 HFD APOE cKO and D374Y CD5L mice (Fig. 6c).

Next, using only the littermate controls, we compared the gene expression changes between day 10 chow-fed and week 4 HFD-fed control mice, and, hence, in the absence of atherogenic APOB lipoproteins, to determine the effects of HFD alone on the liver. We identified strong overlaps between the littermate controls of both strains, with 463 genes similarly dysregulated (Fig. 6d) and metabolic pathways enriched (Extended Data Fig. 6f). In noticeable contrast to the APOB lipoprotein–induced gene expression changes in APOE cKO and D374Y mice, HFD alone activated a hepatocyte-driven response (Extended Data Fig. 6g), and none of the 32 genes differentially expressed after 4 weeks of HFD in the APOE cKO and D374Y strains was dysregulated by HFD alone, thus indicating that this specific response requires APOB lipoproteins (Fig. 6e).

**Fig. 7:**
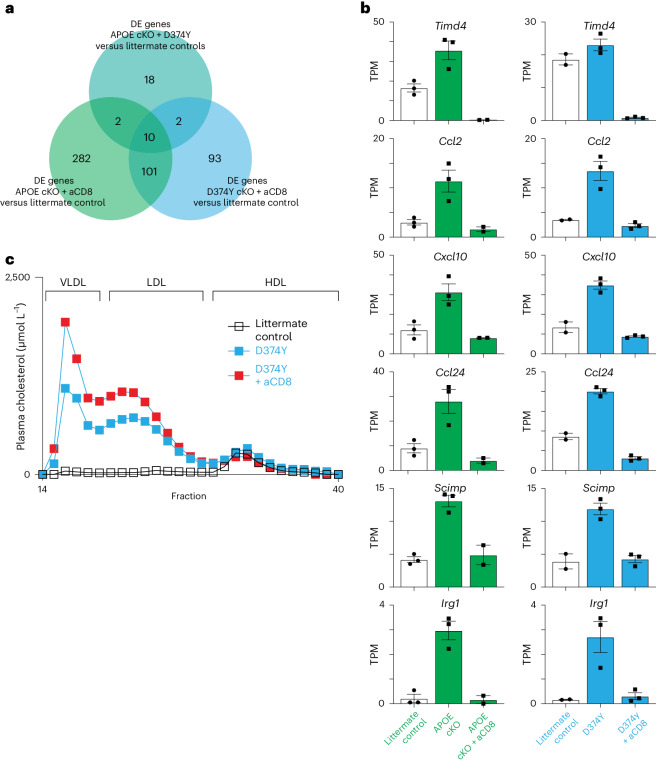
CD8 T cells maintain KC responses to atherogenic dyslipidemia. **a**, Venn diagram representing overlapping genes among differentially regulated genes common to dyslipidemic female APOE cKO and D374Y mice after 4 weeks of HFD, differentially regulated genes in dyslipidemic APOE cKO mice after 4 weeks of HFD and treated with anti-CD8 antibody relative to PBS-treated mice and D374Y mice after 4 weeks of HFD and treated with anti-CD8 antibody relative to PBS-treated mice, as determined by mRNA-seq. **b**, Transcript per million (TPM) of selected KC-specific genes showing downregulation after 4 weeks on an HFD and treatment with anti-CD8 antibody (APOE cKO *n* = 3 versus *n* = 3 versus *n* = 2 and D374Y *n* = 2 versus *n* = 3 versus *n* = 3). All plots are mean ± s.e.m. **c**, Plasma lipoprotein fractionation profiles (µmol L^−1^). All curves were calculated as an average of plasma pooled from female mice (*n* = 5–6). DE, differentially expressed; VLDL, very-low-density lipoprotein.

**Extended Data Fig. 7:**
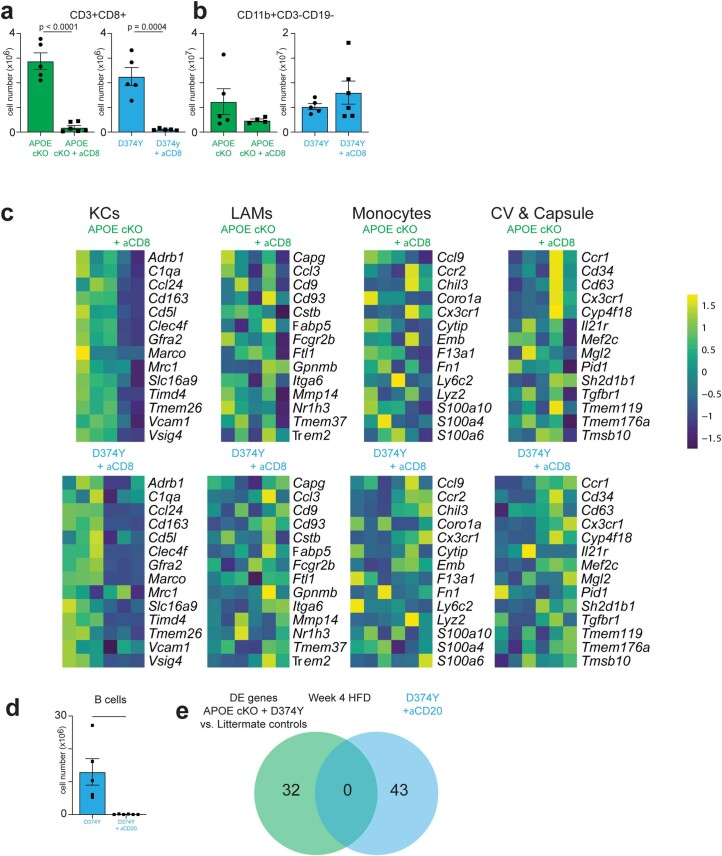
B cells are not required for the KC response to atherogenic dyslipidemia. **a,b**, Absolute numbers of Spleen CD8 T cells (**a**) and CD11b + CD3-CD19- myeloid (**b**) in female APOE cKO and D374Y mice treated with anti-CD8 antibody relative to PBS treated mice as determined by flow cytometry. Mice were maintained on HFD for 4 weeks following Tamoxifen administration. CD3 + CD8: n = 5 APOE cKO and n = 5 APOE cKO + αCD8 mice; n = 5 D374Y and n = 6 D374Y + αCD8 mice. CD11b + CD3-CD19-: n = 5 APOE cKO and n = 4 APOE cKO + αCD8 mice; n = 5 D374Y and n = 6 D374Y + αCD8 mice. **c**, Heatmaps of bulk mRNA seq of whole liver showing variations in the expression of core identity genes for KCs, LAMs, monocytes, CV and capsular macrophages following CD8 T cell depletion in both strains. **d**, Absolute numbers of Spleen B cell (CD19 + B220 + ) in D374Y female mice treated with anti-CD20 antibody relative to PBS treated mice as determined by flow cytometry. Mice were maintained on HFD for 4 weeks following Tamoxifen administration (n = 4 D374Y and n = 6 D374Y + αCD20 mice). **e**, Overlap of genes upregulated in both APOE cKO and D374Y mice versus those upregulated in D374Y mice but treated with anti-CD20 antibody, in all cases after 4 weeks of high-fat diet following tamoxifen-induction. All plots are ± SEM. Statistical analysis was performed with two sided *t*-test (**a**,**b** and **d**).

CD8 T cells are necessary for maintaining adipose macrophages^28^ and KCs^29^. We antibody depleted CD8 T cells during the 4-week HFD regime (Extended Data Fig. 7a) as an alternative approach to clodronate-mediated depletion. Splenic macrophages were unaffected by this treatment (Extended Data Fig. 7d), but we did observe specific downregulation of the KC gene expression program in APOE cKO and D374Y strains but not of other liver myeloid cell types (Extended Data Fig. 7c). Only 10 of the 28 conserved genes remained upregulated in both APOE cKO and D374Y mice after CD8 depletion (Fig. 7a,b). As an additional control, we used anti-CD20 in D374Y mice to delete B cells (Extended Data Fig. 7d), as these can also uptake mCherry-APOB but are not known to deplete KCs. In contrast to anti-CD8 treatment, no loss of the conserved gene expression program was now observed (Extended Data Fig. 7e). Finally, as in the clodronate administration experiment, plasma APOB lipoprotein levels also increased upon loss of CD8 T cells in D374Y mice relative to control treated (Extended Data Fig. 7c).

## Discussion

APOB lipoproteins have a decisive pathological role in the development of atherosclerosis and can also contribute to MASLD^1,3^. To date, whether responses to the acute onset of an atherogenic lipid profile exist outside the vasculature has not been determined. In the present study, we generated mouse models to investigate the dynamics of how APOB lipoproteins can accumulate in the liver based on inducible loss-of-function or gain-of-function alleles that mimic human FH. Our experimental approach allows us to abruptly induce an atherogenic lipid profile in adult mice that have previously been untouched by this dyslipidemic insult.

KCs rapidly responded and had key functional responsibility for signaling a systemic response at the onset of atherogenic dyslipidemia. This conclusion is based on multiple lines of evidence. First, bulk mRNA-seq analysis of the liver revealed known KC genes being differentially regulated upon transition to dyslipidemia. Second, through use of the mCherry-APOB reporter mouse, KCs were shown to recognize APOB lipoproteins in the context of atherogenic dyslipidemia. KCs also dominated the landscape of an unbiased scRNA-seq analysis of mCherry-APOB-positive immune cells. Deleting KCs directly through clodronate liposomes almost completely abolished the whole-liver transcriptional response to acute dyslipidemia. Indirectly deleting KCs via anti-CD8 administration also extinguished the KC-specific inflammatory response, whereas control anti-CD20 did not. Finally, KCs were shown to directly uptake labeled APOB lipoproteins in vivo.

One subset of KCs was enriched for pro-inflammatory mediators, such as *Il1b*, *Tnf* and *Ccl4*. One possibility that requires further experimentation is that this inflammatory subset represents a differentiation step occurring after APOB lipoprotein uptake. KCs have recently been subdivided into KC1 and KC2 populations, with KC2 reportedly regulating metabolism^30,31^, although this interpretation has been challenged^19,32^. We only observed *Esam* expression, used to define KC2, in the endothelial cluster. Hence, APOB lipoprotein responses by KCs do not seem to involve this claimed KC2 subset.

Notably, deleting KCs markedly increased plasma cholesterol and triglyceride levels in D374Y mice and decreased cholesterol and triglyceride content in the livers of D347Y and APOE cKO mice, respectively. KCs in rabbits and rats are known to take up considerable amounts of circulating LDL^4–6^. Taken together with observations from *Apoe*^*−/−*^ mice in the specific context of HFD and hereditary hemochromatosis^7^, our results further underscore the critical role that KCs play in regulating plasma lipoprotein levels. Taking all this evidence together, liver uptake of APOB lipoproteins is not the sole privilege of hepatocytes.

KCs have a potentially ambivalent function: they express and secrete pro-inflammatory and anti-inflammatory factors into plasma but, at the same time, retain circulating pro-atherogenic APOB lipoproteins. In this sense, KCs are operating as hepatic sentinels of atherosclerosis initiation. Hepatic KC responses may further modulate plaque responses at distant sites. In line with this, we observed increases in systemic levels of secreted proteins that can both promote (CD5L^27^) and prevent (IL18BP^33^) atherosclerosis. This raises questions regarding the overall long-term effects of KCs on atherosclerosis development. Such questions will be difficult to address. However, individually targeting these factors in KCs may provide insight. One possibility is that there are multiple subsets of liver KCs, similar to aortic phagocytic cells, that respond to elevated levels of APOB lipoproteins^34^. However, it is possible that there is a cell type outside of the liver that is sensitive to clodronate depletion that also contributes to the production of CD5L and IL18BP in plasma.

Subjecting our mice to a sustained HFD over many weeks revealed that the strains had considerable convergence in whole-liver transcriptional response, dominated by genes associated with immune function, thereby underlining the inflammatory nature of chronic steatosis. This prominent inflammatory signal is specific to hypercholesterolemic APOE cKO and D374Y strains, as littermate controls did not similarly respond to an HFD. Noticeably, the APOB lipoprotein response was centered on KCs, whereas the HFD-only response was hepatocyte dominated. These results are consistent with a recent report indicating that macrophages play a non-inflammatory role in mouse models of non-alcoholic steatohepatitis that also lacked an atherogenic dyslipidemic component^35^. In agreement with this, liver function testing and standard histology did not reveal this APOB lipoprotein response. Hence, the initiation of atherogenic dyslipidemia seems to result in hepatic responses that are ‘silent’ to traditional measures of liver health.

APOE also has additional properties beyond lipoprotein transport, including an anti-inflammatory function^18,36^ and restraining germinal center formation^18,37^, and human alleles of APOE are implicated in the risk of developing multiple complex diseases, including cardiovascular disease^38^. The benefit of comparing both APOE cKO and D374Y mice is that it allows for broad conclusions as to whether atherogenic dyslipidemia or, rather, gene-specific effects are driving phenotypes. Approximately half of all genes that were upregulated in both strains at 8 weeks, 12 weeks and 20 weeks of HFD were positively correlated with human liver PCSK9 expression levels. Similarly, the mouse KC genes that are dysregulated in response to atherogenic dyslipidemia are also enriched in human KCs. Our approach of combining two inducible dyslipidemic strains may, therefore, be suitable as a preclinical model for understanding how perturbations in lipoprotein handling can lead to systemic responses. Indeed, mouse models have proven very useful for studying atherosclerosis^39^. However, the strains described here, as well as legacy *Apoe*^*−/−*^ and *Ldlr*^*−/−*^ mice, remain models of FH and, thus, likely represent the far end of the spectrum of disease states caused by elevated levels of APOB lipoproteins.

In summary, we created an experimental platform that allows for the discovery of how atherogenic dyslipidemia can initiate and sustain tissue damage. By using these tools, we identified liver KCs as key mediators of the hepatic response at the onset of atherosclerosis. Further investigation using these strains will likely reveal additional organs that pathologically respond to APOB lipoprotein-mediated dyslipidemia.

## Methods

### Ethical approval statement

The Stockholm Board for Animal Ethics approved the experimental protocols for the mouse studies. All human protocols were approved by the Human Research Ethics Approval Committee of Stockholm, and written informed consent was obtained from all participants according to the Declaration of Helsinki.

### Mice and experimental diets

The APOE cKO mice were described previously^18,37^ and were maintained on the C57BL/6 genetic background, and mice aged 10–12 weeks were used. All mice were housed in a specific pathogen-free vivarium at the Karolinska Institutet. The light/dark period was 12 h/12 h, and mice were kept under standard temperature and humidity conditions (20–22 °C and 45–55% relative humidity). All mice had ad libitum access to food and water. Breeding mice were fed chow diet R36 (12.6 megajoules per kilogram (MJ kg^−1^), 18% protein and 4% fat; Lantmännen). Experimental mice received chow diet (R70, Lantmännen, 12.5 MJ kg^−1^, 14% protein and 4.5% fat) or HFD (R638, Lantmännen, 15.6 MJ kg^−1^, 17.2% protein, 21% fat and 0.15% cholesterol) as stated in each experiment. Female mice were predominately used as they are thought to be more susceptible to atherosclerosis at early timepoints. Male mice were included in the mRNA-seq of weeks 8, 12 and 20 of HFD diet regimes so that the 72 genes upregulated at all timepoints in both strains can be applicable to both male and female genders.

### Generating hPCSK9 D374Y and mCherry-APOB mouse strains

We created a conditionally activated hPCSK9 D374Y gain-of-function mouse model by inserting D374Y mutated hPCSK9 into the *Rosa26* locus. The ROSA26 gene-targeting vector was constructed from genomic C57BL/6N mouse strain DNA (genOway). PCSK9 D374Y human sequence was inserted downstream of a lox-STOP-lox cassette. Knock-in insertion within the ROSA26 locus is done within ROSA26 intron 1, in anti-sense orientation. Homology arms cover 2.8 kb of ROSA26 intron 1. When the floxed STOP cassette is removed by CRE recombinase, human PCSK9 D374Y expression is driven by the CAG promoter. The linearized targeting vector was transfected into C57BL/6 embryonic stem cells (ESCs) (genOway). Proper integration of the knock-in vector was assessed by a variety of PCR screening, including PCR covering the mutant PCSK9 cDNA and the homology arms and PCR with primers hybridizing within the homology arm and upstream/downstream of the homology arm. Integration was validated by Southern blot, and the integrity of the locus was further assessed by sequencing of the whole recombined locus, including the expression cassette and the homology arms. The *ROSA2*^*PCSK9D374Y*^ mice were crossed with *ROSA26*^*CreERt2*^ mice^40^, creating *ROSA26*^*CreERt2/PCSK9D374Y*^ experimental mice. Littermates without the D374Y insert (*Rosa26*^*CreERt2/+*^ *or Rosa26* ^*CreERt2/ CreERt2*^) were always used as controls.

For the knock-in insertion of mCherry into exon 2 downstream of the *Apob* signal peptide in exon 1, a flexible linker ((GGGGS)×3) was inserted between the mCherry sequence and the *Apob* sequence encoding for its mature form. The targeting vector contains the 5′ part of ApoB mouse exon 2, mCherry coding sequence followed by the linker and the neomycin resistance cassette, flanked by loxP sites, within ApoB intron 2. Homology arms are mouse genomic sequences encompassing 3 kb upstream and 2.1 kb downstream of exon 2. G-418-resistant ESC clones were subsequently validated by PCR, using primers hybridizing within and outside the targeted locus, and Southern blot, to assess the proper recombination event on both sides of the targeted locus. Recombined ESC clones were microinjected into C57BL/6 blastocysts and gave rise to male chimeras with a substantial ESC contribution. The *Apob*^*mCherry/+*^ mice were bred with C57BL/6 Cre-deleter mice to remove the neomycin cassette between exon 2 and exon 3 to generate heterozygous mice carrying the reporter allele. These *Apob*^*mCherry/+*^ mice were then bred to homozygosity and further crossed with *ROSA26*^*CreERt2/PCSK9D374Y*^ to create *Apob*^*mCherry/mCherry*^*ROSA26*^*CreERt2/PCSK9D374Y*^ and *Apob*^*mCherry/mCherry*^*ROSA26*^*CreERt2/ CreERt2*^ littermate controls.

### Induction of dyslipidemia

Mice aged 10–14 weeks were induced with oral tamoxifen as previously described^18^. We administered 9 mg of tamoxifen dissolved in 150 µl of peanut oil + 10% ethanol, via single-dose oral gavage, to experimental mice and their littermate controls. The induction efficiency was evaluated by measuring the plasma cholesterol levels. Occasional APOE cKO and D374Y mice that did not show the expected elevation in cholesterol levels were excluded from the study.

### Plasma lipid analyses, composition and ELISA

Blood was drawn via cardiac puncture, transferred to EDTA-coated tubes and centrifuged at room temperature for 5 min at 500*g*. Plasma was separated and stored at −80 °C for further analyses. Plasma total cholesterol and triglycerides were measured with enzymatic colorimetric kits (Randox) according to the manufacturer’s instructions.

Phospholipids (PLs) were evaluated with a Phospholipids C Kit (Fujifilm, Wako Diagnostics). Plasma-free (non-esterified) fatty acid concentrations were assessed by an enzymatic colorimetric method (NEFA-HR^2^, Wako Chemicals). Plasma concentration of glycerol was determined by an enzymatic colorimetric assay (Free Glycerol FS, Diagnostic Systems GmbH). Lipoprotein fractionation was performed for plasma pools of 4–6 mice by fast performance liquid chromatography^41^. The concentration of cholesterol, triglycerides and PLs in each fraction was computed by enzymatic methods using a CHOD-PAP Kit (Roche Diagnostics), a GPO-PAP Kit (Roche Diagnostics) and a Phospholipids C Kit (Fujifilm, Wako Diagnostics), respectively.

AST, ALT, albumin, globulin and bilirubin were tested in plasma using the clinical chemistry analyzer Samsung PT10V.

The concentration of hPCSK9 was measured in tail vein blood by using a Human PCSK9 Quantikine ELISA Kit (R&D Systems) according to kit instructions. The lower limit of quantification (LLOQ) of hPCSK9 in mouse plasma was 625 pg ml^−1^. Mouse CD5L and IL18PB in plasma were determined using dedicated ELISA kits (CD5L: Invitrogen, EM15RB, LLQQ = 8.19 pg ml^−1^; IL18pb: Abcam, ab254509, LLQQ = 468.75 pg ml^−1^).

### Quantification of liver lipids

Liver lipids for the day 10 timepoint were extracted according to the Folch method, as previously described in detail^42^. In brief, liver lipids were extracted by adding 5 ml of Folch solution (chloroform: methanol—2:1 v/v) to 100 mg of snap-frozen samples. After serial drying steps, including adding 1 ml (0.9% NaCl) to separate the phases and adding 1 ml of 1% Triton X-100 in chloroform, lipids solubilized in 0.5 ml of water were obtained. We corrected the total lipids by the protein content in liver though the modified Lowry microassay in plate protocol, with a D Protein Assay Kit (Bio-Rad), with a protein concentration range of 5–250 µg ml^−1^. Liver cholesterol and triglycerides for timepoints other than day 10 (4 weeks and 8 weeks on an HFD) and for mice treated with clodronate were extracted from liver tissues and quantified using dedicated kits (cholesterol: Abcam 65359, LQQ: 1 μg per well; triglycerides: Abcam 65336, LQQ: 1 nmol per well) following the instructions provided. Final lipid concentrations were adjusted for the protein content of each sample (Pierce BCA Protein Assay Kit, Thermo Fisher Scientific, 23225).

Plasma cholesterol and triglyceride concentrations in the total lipids were determined using CHOL2 and TRIGL kits (Roche Diagnostics). CHOL2 has a measuring range of 3.86–800 mg dl^−1^, and TRIGL has a measuring range of 8.85–885 mg dl^−1^.

### Histology

Hematoxylin and eosin staining was performed on 8-μm-thick, 4% Zn-formaldehyde-fixed, paraffin-embedded liver sections. Sections were deparaffinized in Histolab Clear and rehydrated in gradually decreasing ethanol solutions (99%, 95% and 70%). They were then stained with hematoxylin (H-3404, Vector Labs) and eosin (01650, Histolab), dehydrated in ethanol and xylene and mounted in Pertex (00801, Histolab). Slides were scanned using a VS200 slide scanner (Olympus), and pictures were acquired using OlyVIA version 3.4.1 software.

Oil Red O (ORO) was prepared as previously described^43^. Fresh liver and heart tissues were frozen in Tissue-Tek OCT (45830, Histolab) and then sectioned using a Microm HM560 cryostat. Sections of liver (8 μm) and aortic root (10 μm) were then fixed in 4% paraformaldehyde for 10 min and incubated with a filtered ORO solution (0.6 g of ORO, O0625, Sigma-Aldrich, dissolved in 60 ml of isopropanol and 40 ml of distilled water) for 20 min. After rinsing with tap water, hematoxylin was applied for 1 min. Hematoxylin in excess was then washed out with water, and slides were mounted in Kaiser’s glycerin gelatin. Pictures were acquired with a Leica DMRB light microscope.

### Immunofluorescence confocal microscopy

Next, 10-µm-thick sections of liver were thaw mounted to slides that were subsequently fixed with ice-cold acetone or 4% paraformaldehyde for 10 min and stored at −20 °C. Slides were thawed, and sections were incubated in washing buffer TBST (1× Tris-buffered saline with 0.1% Tween 20) for 5 min and then blocked with 5% BSA for 30 min. Primary antibodies against CD68 (clone MCA1957, Serotec, 1:10,000) and F4/80 (30325S, Cell Signaling Technology, 1:400) were incubated at 4 °C overnight. For CD68, the secondary antibody (biotinylated anti-goat IgG (Vector Labs, 1:300)) was incubated first for 1 h at room temperature and then for 1 h with streptavidin-DyLight 647 (Vector Labs, 1:300). For F4/80, DyAlexa 488 anti-rabbit (1:300) was incubated for 1 h at room temperature. Slides were then incubated with DAPI (Invitrogen, 1:50,000) for 20 min. The sections were washed 3 × 3 min in washing buffer after each incubation step. The slides were then mounted with fluorescent mounting media (Dako). Negative control stainings were performed by omitting the primary antibodies from the protocol. Immunofluorescence images were taken with a Nikon Ti-2E confocal microscope using NIS Elements software.

### Immunohistochemistry

Immunohistochemical labeling of CD5L was obtained from 8-µm-thick frozen liver sections, fixed in 4% paraformaldehyde for 10 min. Endogenous peroxidase was quenched with 0.3% H_2_O_2_ for 15 min, and slides were subsequentially incubated with avidin (15 min) and biotin (15 min). After blocking for 30 min with 5% horse serum, the slides were incubated with anti-CD5L antibody (Abcam, ab45408, 1:100) overnight at 4 °C. An anti-rabbit biotinylated secondary antibody (BA-1000, Vector Labs, 1:200) was added on the sections for 30 min at room temperature. Slides were incubated with avidin-biotin-complex-PO (PK-6100, Vector Labs) for 30 min and developed in DAB (5 min, room temperature, SK-4100, Vector Labs). Finally, sections were stained with hematoxylin and mounted with Pertex. Slides were scanned with a VS200 slide scanner (Olympus), and pictures were acquired using OlyVIA version 3.4.1 software.

For immunohistochemistry of adipose tissue, visceral (peri-gonadal) white adipose tissue was dissected and fixed for 48 h in 10% formalin solution (Sigma-Aldrich, HT501128) at room temperature. The fixed tissues were imbedded in paraffin, sectioned at 5 μm and mounted on glass slides. For immunostaining, slides were deparaffinized and rehydrated, and antigen retrieval was performed by boiling the slides in citrate buffer (10 mM citrate acid, 0.05% Tween 20, pH 6.0) in a microwave, followed by cooling overnight. Antibody labeling was performed using an F4/80 rabbit monoclonal antibody (D4C8V, Cell Signaling Technology, 1:100) together with the anti-rabbit HRP-DAB Cell and Tissue Staining Kit (1545297, R&D Systems) according to the manufacturer’s protocol. To show tissue morphology, slides were subsequently stained with hematoxylin and eosin, dehydrated, mounted with Pertex (00840, Histolab) and imaged using a Leica ICC50W bright-field microscope at ×40 magnification.

### qPCR analysis of macrophage marker CD68 in adipose tissue

Visceral (peri-gonadal) white adipose tissue was dissected and lysed using 1 ml of QIAzol and 5-mm stainless steel beads (both Qiagen) in a TissueLyser at 30 Hz for 3 min. RNA was isolated by chloroform extraction followed by the RNAeasy Mini Kit and the RNAse-free DNase Set (74004 and 79254, Qiagen). RNA was eluted using RNase-free water, and concentration was measured using NanoDrop. Then, 1 μg of RNA was reverse transcribed using SuperScript III First-Strand Synthesis SuperMix (11752-050/250, Invitrogen), and qPCR was performed using TaqMan Fast Advanced Master Mix, MicroAmp Fast Optical 96-Well Reaction Plates (4444556 and 4346906, Applied Biosystems) and TaqMan primers for *mCd68* (Mm03047343_m1) and mouse *mTBP* (Mm00446971_m1).

### Western blot

Liver total proteins were extracted with RIPA lysis buffer, and protein concentrations were determined with a BCA protein concentration assay kit (Pierce BCA Protein Assay Kit, Thermo Fisher Scientific, 23225). Under reducing conditions, 10% SDS-PAGE was used to separate proteins, which were subsequently transferred onto PVDF membranes and blocked with 5% non-fat milk at room temperature for 1 h. The membranes were then incubated with primary antibody solutions (LDLR, LSBio, 1:500; β-actin, Sigma-Aldrich, 1:2,000; mCherry, Abcam, 1:1,000; albumin, Thermo Fisher Sciedntific, 1:600) overnight at 4 °C. The next day, the membranes were incubated with HRP-conjugated secondary antibodies at room temperature for 45 min and, after washing with TBST, were developed using a high-sensitivity ECL system.

### Bulk sequencing and scRNA-seq

Mice were euthanized with carbon dioxide and perfused by infusing PBS via the left ventricle. A small sample from one liver lobe was taken, carefully avoiding the gall bladder. Livers were stored in RNALater (Qiagen) at −20 °C until RNA extraction. The livers were solubilized in QIAzol lysis reagent using the TissueLyser, and extracted RNA was isolated to the upper fraction by chloroform. Purification was performed with the RNeasy Mini Kit including on-column DNAse treatment, using a QIAcube robot. RNA was selected using a Poly(A) RNA Selection Kit (Lexogen), and sequencing libraries were prepared with Lexogen QuantSeq version 2. DNA fragments 200–800 bp for RNA-seq were selected. Cluster generation and sequencing was carried out by using an Illumina HiSeq version 4 system with a read length of 50 nucleotides (single-read) or NovaSeq with a read length of 150 nucleotides (paired-end) and aligned to the mouse transcriptome genome assembly version of July 2007 (NCBI37/mm9) using TopHat version 1.4.1 or mouse genome assembly version of December 2011 (GRCm38/mm10) using STAR 2.4.2a^38^ in the transcriptome-guided alignment mode. Reads per gene were counted using HTSeq version 0.5.3 with the overlap resolution mode set to union. Differential expression of mRNA was analyzed using DeSeq2 software at default settings, with a false discovery rate set at 0.1 for all experiments, as previously described^44^.

For scRNA-seq, live CD45^+^mCherry^+^ cells were sorted from the liver. A small sample from one lobe was cut into small pieces and digested in 0.2 mg ml^−1^ collagenase IV for 30 min at 37 °C in RPMI 1640. The cell suspension was passed through an 18-gauge syringe 10 times to remove any clumps and then a 70-µm cell strainer. The cell suspension was then washed with FACS buffer and stained with CD45 (V500 30-F11, 1:500), LIVE/DEAD Aqua (Invitrogen) and TotalSeq-A0301 anti-mouse Hashtag 1 antibody (BioLegend) to determine liver cells. Cells were sorted on a Sony SH800S cell sorter and processed immediately for GEM generation and barcoding on a 10x Chromium using Next GEM 3′ version 3.1 reagents (10x Genomics), followed by sequencing and processing on Cell Ranger. Data analysis was performed in R using Seurat version 4 (ref. ^45^) with the gene *Gm42418* removed from the dataset.

### Human samples and data

Liver biopsies were obtained from patients undergoing aortic valve and/or ascending aortic surgery as part of the Advanced Study of Aortic Pathology (ASAP)^46^. Gene expression was measured in liver samples (*n* = 261) using the Human Transcriptomic Array 2.0 (Affymetrix), as previously described^47^. Samples were hybridized and scanned at the Karolinska Institutet Affymetrix core facility, and raw data were pre-processed using Robust Multichip Average (RMA) normalization as implemented in the Affymetrix Transcriptome Analysis Console (TAC). Expression values were log_2_ transformed as part of the RMA normalization.

The Single Cell Type section of the Human Protein Atlas contains data from human scRNA-seq experiments retrieved from published studies based on healthy human tissues^20,21^. Transcriptomic expression data for 76 consensus single cell types were obtained from the Human Protein Atlas for the two sets of conserved genes between mouse and human. Analyses were performed using R statistical software^48^, and the ‘pheatmap’^49^ package was used to visualize expression profiles. The dendrograms were obtained by hierarchical clustering of distances based on gene expression levels for all single cell types, using Ward’s criterion.

### Detection of cell types by flow cytometry

Spleens were ground with syringe plungers and prepared as single-cell suspensions by pressing through sterile 70-µm mesh size cell strainers. Cells were stained with conjugated antibodies on ice for 30 min. Liver was processed as described above for scRNA-seq. The following antibody clones were used: CD3ε (PB 500A2, 1:2,000), CD8 (FITC 53-6.7, 1:500), CD4 (BV750 GK1.5, 1:500), CD45 (V500 30-F11, 1:500), CD19 (PE eBio1D3, 1:500), B220 (APC-Cy7 RA3-6B2, 1:500), CD172 (BV711 P84, 1:500), Ly6G (PB 1A8, 1:250), CD11b (PerCP/Cyanine5.5 M1/70, 1:500), CD11c (APC N418, 1:500), MHCII (AF700 M5/114.15.2, 1:500), F4/80 (BV510 BM8, 1:250) and TIMD4 (PerCP-eF710 54, 1:500). Immune cell populations were defined as liver KCs (CD3^−^CD19^−^Ly6C^−^F4/80^+^TIMD4^+^CD64^+^), liver plasmacytoid dendritic cells (CD19^−^B220^+^CD172^+^), liver neutrophils (F4/80^−^CD11b^+^Ly6G^+^), liver dendritic cells (CD11c^+^MHCII^+^), liver B cells (CD19^+^B220^+^), spleen CD8 T cells (CD3^+^CD4^−^CD8^+^) and spleen B cells (CD45^+^CD19^+^B220^+^). The samples were acquired with Northern Lights or Aurora spectral flow cytometers (Cytek) with SpectroFlo software and analyzed with FlowJo software version 10.8.1.

### Antibody administration and lymphocyte cell depletion

CD8 cells were depleted with anti-CD8a antibody (rat IgG2b anti-mouse CD8α YTS 169.4, Bio X Cell) or anti-mouse CD20 (mouse Ig2c anti-mouse CD20 MB20-11, Bio X Cell). Mice were injected with 250 µg of antibody in 150 µl of PBS (anti-CD8) or 100 µg of antibody in 200 µl (anti-CD20) or similar volume of PBS intraperitoneally once weekly. Five days after the first injections, tamoxifen was administered via oral gavage, and the mice began receiving an HFD. This diet and weekly injections continued for 4 weeks.

### Clodronate administration and liver macrophage depletion

Clodronate liposomes or Dil liposomes control containing PBS (LIPOSOMA) were given via intravenous tail vein injections (100 µl of 5 mg ml^−1^ suspension). For short-term clodronate treatments, mice received a total of three injections on days 0, 4 and 8 from tamoxifen administration. For long-term clodronate treatment, mice received one injection of clodronate per week for a total of 8 weeks while being subjected to an HFD.

### Lipoprotein labeling and/or administration

LDL lipoproteins were purified from plasma of D374Y mice using an LDL/VLDL Purification Kit (Cell Biolabs) and labeled with AF-488 using an Alexa Fluor Antibody Labeling Kit (Life Technologies). Then, 100 μl of the obtained solution (AF-488 LDL = 5.45 mg ml^−1^) was administered intravenously to a dyslipidemic APOE cKO mouse induced with tamoxifen 10 d prior. PBS was administered as control to a different mouse. Both mice were euthanized 30 min after receiving the injections.

Next, 100 μl of plasma from three different mCherry-APOB D374Y mice previously subjected to 8 weeks of high-fat feeding was pulled together and administered intravenously to an APOE cKO mouse induced with tamoxifen 10 d prior. The same amount of plasma from three different D374Y mice also subjected to 8 weeks of high-fat feeding was given to another induced APOE cKO mouse as control. Mice were euthanized 30 min after injection.

### Statistical analysis

Data are presented as mean ± s.e.m. A two-tailed unpaired Student’s *t*-test was used for comparing two experimental groups. A two-tailed ANOVA followed by Fisher’s least significant difference was used for comparisons of more than two groups. The Pearson correlation coefficient was used to compare transcript levels in human livers. *P* < 0.05 was considered significant. Statistical analyses were performed using GraphPad Prism 9 software. Figures were assembled in Adobe Illustrator.

### Reporting summary

Further information on research design is available in the Nature Portfolio Reporting Summary linked to this article.

### Supplementary information

Reporting Summary

### Source data

Source Data Fig. 1 Source data for all figures and extended data.

Source Data Extended Data Fig. 4 Full western blot.

## Data Availability

The mRNA-seq (GSE254879) and scRNA-seq (GSE254971) data are available at the Gene Expression Omnibus. Source data are provided with this paper.

## Code availability

Processing of scRNA-seq can be found at https://github.com/yuyzha/Malin-Group_NCR.
