# Supplementary material for: Kupffer cells dictate hepatic responses to the atherogenic dyslipidemic insult
Source: Nat Cardiovasc Res. 2024 Mar 11;3(3):356–71. doi: 10.1038/s44161-024-00448-6 (PMC11358021; doi:10.1038/s44161-024-00448-6)
Supplement: Supplementary file 1 — Reporting Summary [file 44161_2024_448_MOESM1_ESM.pdf]

nature portfolio

Corresponding author(s): Stephen Malin
Last updated by author(s): Mar 2, 2024

Reporting Summary

Nature Portfolio wishes to improve the reproducibility of the work that we publish. This form provides structure for consistency and transparency in reporting. For further information on Nature Portfolio policies, see our Editorial Policies and the Editorial Policy Checklist.

Statistics

For all statistical analyses, confirm that the following items are present in the figure legend, table legend, main text, or Methods section.

n/a	Confirmed	
☐	☒	The exact sample size (n) for each experimental group/condition, given as a discrete number and unit of measurement
☒	☐	A statement on whether measurements were taken from distinct samples or whether the same sample was measured repeatedly
☐	☒	The statistical test(s) used AND whether they are one- or two-sided<br>*Only common tests should be described solely by name; describe more complex techniques in the Methods section.*
☒	☐	A description of all covariates tested
☐	☒	A description of any assumptions or corrections, such as tests of normality and adjustment for multiple comparisons
☐	☒	A full description of the statistical parameters including central tendency (e.g. means) or other basic estimates (e.g. regression coefficient) AND variation (e.g. standard deviation) or associated estimates of uncertainty (e.g. confidence intervals)
☐	☒	For null hypothesis testing, the test statistic (e.g. F, t, r) with confidence intervals, effect sizes, degrees of freedom and P value noted<br>*Give P values as exact values whenever suitable.*
☒	☐	For Bayesian analysis, information on the choice of priors and Markov chain Monte Carlo settings
☒	☐	For hierarchical and complex designs, identification of the appropriate level for tests and full reporting of outcomes
☐	☒	Estimates of effect sizes (e.g. Cohen's d, Pearson's r), indicating how they were calculated

Our web collection on statistics for biologists contains articles on many of the points above.

Software and code

Policy information about availability of computer code

Data collection	Slides were scanned using a Olympus VS200 slide scanner and pictures were acquired using OlyVIA V3.4.1 software. Immunofluorescence images were taken with Nikon Ti-2E confocal microscope and NIS Elements software. Cells were sorted by a Sony SH800S cell sorter and associated software. Flow cytometry was performed with Cytek Northern Lights or Aurora spectral flow cytometers with Spectroflo software. scRNA-seq was processed on 10X Genomics cell ranger software.
Data analysis	Data analysis was performed in R using Seurat v4 for scRNA-seq. mRNA-seq was aligned to the mouse transcriptome using TopHat v1.4.1 or STAR 2.4.2a. Reads per gene were counted using HTseq v0.5.3 or STAR 2.4.2a and analysed in R using DeSeq2. Heatmaps were made using Pheatmap v1.0.12. Flow cytometry data was analysed by FlowJo v10.8.1. Statistical analysis was performed in GraphPad Prism 9. Figures were assembled in Adobe Illustrator.

For manuscripts utilizing custom algorithms or software that are central to the research but not yet described in published literature, software must be made available to editors and reviewers. We strongly encourage code deposition in a community repository (e.g. GitHub). See the Nature Portfolio guidelines for submitting code & software for further information.

Data

Policy information about availability of data

All manuscripts must include a data availability statement. This statement should provide the following information, where applicable:

- Accession codes, unique identifiers, or web links for publicly available datasets
- A description of any restrictions on data availability
- For clinical datasets or third party data, please ensure that the statement adheres to our policy

The mRNA-seq and scRNA-seq data generated for this study are available at GEO under accession no. GSE254879 and GSE254971

Human research participants

Policy information about studies involving human research participants and Sex and Gender in Research.

Reporting on sex and gender	The clinical samples have been reported elsewhere PMID: 21620721. Sex was self-reported in the questionnaire obtained from patients Male: 194. Female: 67. Sex was not taken into account for the correlative data analysis. Patients were recruited consecutively irrespective of sex.
Population characteristics	The clinical samples have been reported elsewhere PMID: 21620721. Mean age 64.36, minimum age 26, maximum 86. Std deviation 11.826. No covariate analysis was used.
Recruitment	Patients undergoing elective open-heart surgery for ascending aortic repair and/or aortic valve disease at the Karolinska University Hospital, Stockholm. Patients were recruited consecutively.
Ethics oversight	The study was approved by the Human Research Ethics Approval Committee in Stockholm (2006/784-31/1). Written consent was obtained from all patients according to the declaration of Helsinki. Consent has been acquired from human participants to process/share data.

Note that full information on the approval of the study protocol must also be provided in the manuscript.

Field-specific reporting

Please select the one below that is the best fit for your research. If you are not sure, read the appropriate sections before making your selection.

☒ Life sciences ☐ Behavioural & social sciences ☐ Ecological, evolutionary & environmental sciences

For a reference copy of the document with all sections, see nature.com/documents/nr-reporting-summary-flat.pdf

Life sciences study design

All studies must disclose on these points even when the disclosure is negative.

Sample size	A minimum of two biological replicates were used for each comparison. Sample size was determined and limited by availability of mouse litter sizes for each genotype and the sex of the mice, so that sex-matched littermates could be used. The maximum amount of matching mice available were used. No size calculation was used. For human data, Patients underwent elective open-heart surgery for ascending aortic disease and/or aortic valve disease, consecutively included. Liver biopsies were collected from 261 patients with no relation to their aortic-aortic valve disease, no known liver disease and pre-operative liver values with no abnormalities detected. No size calculation was used
Data exclusions	Occasional APOE cKO and D374Y mice that did not show the expected elevation in cholesterol levels were excluded from experimental batches and not evaluated further. For bulk mRNA-seq, principle component analysis of whole transcriptome normalised counts was used to determine inclusion. For the week 8 HFD clodronate analysis, 2 experimental samples were excluded based on incomplete depletion of kupffer cells. For human data, gene correlations were analyzed in all patients with no stratification. No co-variates were included in the analysis.
Replication	No technical replicates are included in the study. All attempts at replication were successful. All individual points represent a single data point from one mouse or human as indicated. Blood lipid analysis has been replicated at least twice to confirm the dyslipidemic phenotype of the mice. HPLC of plasma lipoprotein levels was successfully replicated once. Analysis of hepatic lipids levels was successfully replicated by Oil Red O staining in independent experiments once for every mouse group. For mRNA-seq experiments, biological replicates from independent experiments were combined when possible. The mRNA-seq of APOE cKO or D374Y livers was performed independently of each other. To further replicate the mRNA-seq results, selected secreted factors were measured in plasma by ELISA in independent cohorts. Due to the high sequencing costs, there was no attempt to replicate the scRNA-seq experiment. The flow cytometry of mCherry-APOB was replicated at least once and all attempts were successful. The human analysis was not replicated due to a lack of comparable cohort.
Randomization	Where applicable, mice of the same genotype were randomly assigned to treatment groups.
Blinding	Investigators were not blinded to the genotype of the mice. Sample collection and processing was performed blinded with the exception of

Blinding	plasma analysis, as the dyslipidemic status of individual mice is often apparent in the sample. In no experiment was animal handling, sample processing and data analysis performed by the same investigator. For human data, no patient or analysis stratification was performed.

Reporting for specific materials, systems and methods

We require information from authors about some types of materials, experimental systems and methods used in many studies. Here, indicate whether each material, system or method listed is relevant to your study. If you are not sure if a list item applies to your research, read the appropriate section before selecting a response.

Materials & experimental systems

n/a	Involved in the study
☐	☒ Antibodies
☒	☐ Eukaryotic cell lines
☒	☐ Palaeontology and archaeology
☐	☒ Animals and other organisms
☐	☒ Clinical data
☒	☐ Dual use research of concern

Methods

n/a	Involved in the study
☒	☐ ChIP-seq
☐	☒ Flow cytometry
☒	☐ MRI-based neuroimaging

Antibodies

Antibodies used

CD3ε (BV785 145-2C11 Biolegend cat. no.100355), CD8 (FITC 53-6.7 BD Biosciences cat. no.553031 lot no.1029933), CD4 (BV750 GK1.5 Biolegend cat. no.100467 lot no.B341266), CD45 (V500 30-F11 BD Biosciences cat. no.561487 lot no.1131052), CD19 (PE eBio1D3 eBioscience cat. no.12-0193-85 lot no.4277554), B220 (APC-Cy7 RA3-6B2 Biolegend cat. no.103224 lot no.B321245), CD172 (BV711 P84 BD Biosciences cat. no.740766 lot no.1263446), Ly6G (PB 1A8 Biolegend cat. no.127612 lot no.B336505), CD11b (PerCP/Cyanine5.5 M1/70 Biolegend cat. no.101228 lot no.B326435), CD11c (APC N418 Biolegend cat. no.117310 lot no.B331091), MHCII (AF700 M5/114.15.2 Biolegend cat. no.107622 lot no.B350375), F480 (BV510 BM8 Biolegend cat. no.123135 lot no.B305280), TIMD4 (PerCP-eF710 54 invitrogen cat. no.46-5866-82 lot no.2178334), Ly6C (BV650 HK1.4 Biolegend cat. no.128049 lot no.B329651), CD64 (BV421 X54-5/7.1 Biolegend cat no.139309).

Cell depletion antibodies: CD8 cells were depleted with anti-CD8a antibody (Rat IgG2b anti-mouse CD8α YTS 169.4, BioXcell cat. no.BE0117 lot no.728419M1) or anti-mouse CD20 (Mouse Ig2c anti-mouse CD20 MB20-11 BioXcell cat. no.BE0356 lot no.MB20-11).

Western blot: LDLR: LifeSpan Biosciences, unconjugated, cat n. LS-C146979, lot no.203769/100. Anti-β-Actin: AC-15, Sigma-Aldrich, unconjugated, cat n. A1978 lot no.088M4804V. Serum albumin: Bioss, unconjugated, cat n. bs-2256R, lot no.BC2221037. Anti-mCherry: abcam, unconjugated, cat n. ab167453, lot no. GR3297302.

Confocal microscopy and immunohistochemistry: CD68 (MCA1957, Serotec, clone FA-11), F4/80 (30325S, CellSignaling clone D4C8V) , CD5L (abcam, ab45408, clone CT-2 lot no.GR180210-43).

Validation

Cell depleting antibodies (anti-mouse CD8α and anti-mouse CD20) were validated by flow cytometry for loss of relevant cell type and confirmed in the manuscript. According to the manufacturer website, both antibodies have been previously validated by numerous studies. Flow cytometry antibodies were validated by information available on company websites and our experience in routine flow cytometry analysis of lymphoid organs.

FACS
CD3ε BV785 145-2C11 Biolegend Catalogue number: 100355 https://www.biolegend.com/fr-ch/products/brilliant-violet-785-anti-mouse-cd3epsilon-antibody-12081?GroupID=BLG248
CD8 FITC 53-6.7 BD Biosciences Catalogue number: 553031 https://www.bdbiosciences.com/en-eu/products/reagents/flow-cytometry-reagents/research-reagents/single-color-antibodies-ruo/fitc-rat-anti-mouse-cd8a.553031
CD4 BV750 GK1.5 Biolegend Catalogue number: 100467 https://www.biolegend.com/en-ie/products/brilliant-violet-750-anti-mouse-cd4-antibody-15756
CD45 V500 30-F11 BD Biosciences Catalogue number: 561487 https://www.bdbiosciences.com/en-eu/products/reagents/flow-cytometry-reagents/research-reagents/single-color-antibodies-ruo/v500-rat-anti-mouse-cd45.561487
CD19 PE eBio1D3 eBioscience Catalogue number: 4277554 https://www.thermofisher.com/antibody/product/CD19-Antibody-clone-eBio1D3-1D3-Monoclonal/12-0193-82
B220 APC-Cy7 RA3-6B2 Biolegend Catalogue number: 103224 https://www.biolegend.com/en-ie/products/apc-cyanine7-anti-mouse-human-cd45r-b220-antibody-1938
CD172 BV711 P84 BD Biosciences Catalogue number: 740766 https://www.bdbiosciences.com/en-se/products/reagents/flow-cytometry-reagents/research-reagents/single-color-antibodies-ruo/bv711-rat-anti-mouse-cd172a.740766
Ly6G PB 1A8 Biolegend Catalogue number: 127612 https://www.biolegend.com/en-us/products/pacific-blue-anti-mouse-ly-6g-antibody-6082
CD11b PerCP/Cyanine5.5 M1/70 Biolegend Catalogue number: 101228 https://www.biolegend.com/en-gb/search-results/percp-cyanine5-5-anti-mouse-human-cd11b-antibody-4257?GroupID=BLG10552
CD11c APC N418 Biolegend Catalogue number: 117310 https://www.biolegend.com/en-gb/products/apc-anti-mouse-cd11c-antibody-1813?GroupID=BLG11937
MHCII AF700 M5/114.15.2 Biolegend Catalogue number: 107622 https://www.biolegend.com/fr-ch/products/alexa-fluor-700-anti-mouse-i-a-i-e-antibody-3413?GroupID=BLG4736
F480 BV510 BM8 Biolegend Catalogue number: 123135 https://www.biolegend.com/en-gb/sean-tuckers-tests/brilliant-violet-510-

anti-mouse-f4-80-antibody-8934?GroupID=BLG5319
TIMD4 PerCP-eF710 54 invitrogen Catalogue number: 46-5866-82 https://www.thermofisher.com/antibody/product/TIM-4-Antibody-clone-54-RMT4-54-Monoclonal/46-5866-82
CD64 BV421 X54-5/7.1 Catalogue number: 139309 https://www.biolegend.com/ja-jp/products/brilliant-violet-421-anti-mouse-cd64-fcgammari-antibody-8992?GroupID=BLG8805
Ly6C BV650 HK1.4 Biolegend Catalogue number: 128049 https://www.biolegend.com/ja-jp/search-results/brilliant-violet-650-anti-mouse-ly-6c-antibody-17378?GroupID=BLG7242

Depleting antibody
anti-CD8a YTS 169.4, BioXcell Catalogue number: BE0117 https://bioxcell.com/invivomab-anti-mouse-cd8a-be0117?gad_source=1&gclid=CjwKCAiA5L2tBhBTEiwAdSxJXx5epkE3aN-7m7w-VXba6iapzpQl4RK_4NEfNFqwY92pX4DCs8dxEhoCANsQAvD_BwE
anti-mouse CD20 MB20-11, BioXcell Catalogue number: BE0356 https://bioxcell.com/invivomab-anti-mouse-cd20-be0356

Western Blot
LDLR LifeSpan Biosciences Catalogue number: LS-C146979 https://www.labome.com/product/LifeSpan-Biosciences/LS-C146979.html
β-Actin AC-15 Sigma Catalogue number: A1978 https://www.sigmaaldrich.com/SE/en/product/sigma/a1978
mCherry abcam Catalogue number: ab167453 https://www.abcam.com/en-se/products/primary-antibodies/mcherry-antibody-ab167453
Albumin Bioss Catalogue number: bs-2256R https://www.biossusa.com/products/bs-2256r

Confocal microscopy
and IHC
CD68 FA-11, Serotec Catalogue number: MCA1957 https://www.bio-rad-antibodies.com/monoclonal/mouse-cd68-antibody-fa-11-mca1957.html?f=purified&JSESSIONID_STERLING=4DAD7B299E517EEE0D02D657D7F5F9CC.ecommerce2&evCntryLang=SE-en&cntry=SE&thirdPartyCookieEnabled=true
F4/80 D4C8V CellSignaling Catalogue number: 30325S https://www.cellsignal.com/products/primary-antibodies/f4-80-d4c8v-xp-rabbit-mab/30325
CD5L CT-2, abcam Catalogue number: ab45408 https://www.abcam.com/en-se/products/primary-antibodies/cd5l-ct-2-antibody-ab45408

Confocal microscopy
and IHC
CD68 FA-11, Serotec MCA1957 https://www.bio-rad-antibodies.com/monoclonal/mouse-cd68-antibody-fa-11-mca1957.html?f=purified&JSESSIONID_STERLING=4DAD7B299E517EEE0D02D657D7F5F9CC.ecommerce2&evCntryLang=SE-en&cntry=SE&thirdPartyCookieEnabled=true
F4/80 D4C8V CellSignaling 30325S https://www.cellsignal.com/products/primary-antibodies/f4-80-d4c8v-xp-rabbit-mab/30325
CD5L CT-2, abcam ab45408 https://www.abcam.com/en-se/products/primary-antibodies/cd5l-ct-2-antibody-ab45408

Animals and other research organisms

Policy information about studies involving animals; ARRIVE guidelines recommended for reporting animal research, and Sex and Gender in Research

Laboratory animals	All mice used were aged between 10-14 weeks at the start of the experiment. The APOE cKO mice were maintained on the C57BL/6 genetic background and described previously PMID: 29880490. We created a conditionally activated hPCSK9 D374Y gain-of-function mouse model by inserting D374Y mutated hPCSK9 into the Rosa26 locus and targeting of C57BL/6 embryonic stem cells. The ROSA2PCSK9D374Y mice were crossed with ROSA26CreERt2 mice (PMID: 17251932) creating ROSA26CreERt2/PCSK9D374Y experimental mice. Littermates without the D374Y insert (Rosa26CreERt2/+ or Rosa26 CreERt2/ CreERt2) were always used as controls. mCherry was inserted into exon 2 of Apob. The ApobmCherry/+ was bred with C57BL/6 Cre-deleter mice to remove the neomycin cassette between exon 2 and 3 in order to generate heterozygous mice carrying the reporter allele. These ApobmCherry/+ were then bred to homozygosity and further crossed with ROSA26CreERt2/PCSK9D374Y to create ApobmCherry/mCherry ROSA26CreERt2/PCSK9D374Y and ApobmCherry/mCherry ROSA26CreERt2/ CreERt2 littermate controls. All mice were housed in a specific pathogen-free vivarium at the Karolinska Institute. The light/dark period was 12h/12h, and mice were kept under standard temperature and humidity conditions (20-22°C, 45-55% RH). All mice had ad libitum access to food and water. Breeding mice were fed chow diet R36 (12.6 MJ/kg, 18% protein, 4% fat; Lantmännen, Sweden). Experimental mice received chow diet (R70, Lantmännen, Sweden, 12.5 MJ/kg, 14% protein, 4.5% fat) or HFD (R638, Lantmännen, Sweden, 15.6 MJ/kg, 17.2% protein, 21% fat, 0.15% cholesterol) as stated in each experiment. Littermates controls were used.
Wild animals	The Study did not invovle wild animals
Reporting on sex	Either Male or female mice were used as stated in the manuscript for all experiments with the exception of plasma lipid analysis were mixed male and female samples were combined.
Field-collected samples	The study did not involve field-collected samples
Ethics oversight	The Stockholm board for animal ethics approved the experimental protocols.

Note that full information on the approval of the study protocol must also be provided in the manuscript.

Clinical data

Policy information about clinical studies

All manuscripts should comply with the ICMJE guidelines for publication of clinical research and a completed CONSORT checklist must be included with all submissions.

Clinical trial registration	Not a clinical trial. Clinical samples have been reported elsewhere PMID: 21620721.
Study protocol	Patients undergoing elective open-heart surgery for ascending aortic repair and/or aortic valve disease at the Karolinska University Hospital, Stockholm. Liver samples were collected during surgery PMID: 21620721.
Data collection	Data published in PMID: 24927523 & 20562444. Transcriptomics using HTA Affymetrix. Samples were hybridized and scanned at the Karolinska Institute Affymetrix core facility. Output in (.cel format). Only omics data that have passed the quality control by the manufacturers and providers was included in the analysis. Data was quality-checked by our in-house criteria. Gene expression (Affymetrix Exon array) data has been deposited (https://www.ncbi.nlm.nih.gov/geo/query/acc.cgi?acc=GSE26155).
Outcomes	not applicable.

Flow Cytometry

Plots

Confirm that:

☒ The axis labels state the marker and fluorochrome used (e.g. CD4-FITC).

☒ The axis scales are clearly visible. Include numbers along axes only for bottom left plot of group (a 'group' is an analysis of identical markers).

☒ All plots are contour plots with outliers or pseudocolor plots.

☒ A numerical value for number of cells or percentage (with statistics) is provided.

Methodology

Sample preparation	Spleens were ground with syringe plungers and prepared as single-cell suspensions by pressing through sterile 70 µm mesh size cell strainers. Cells were stained with conjugated antibodies on ice for 30 minutes. For liver, a small sample from one lobe was cut into small pieces and digested in 0.2 mg/mL collagenase IX for 30 min at 37 °C in RPMI 1640. The cell suspension was passed through an 18 g syringe 10 times to remove any clumps and then a 70uM cell strainer.
Instrument	Spectral Flow cytometry was performed with Cytek Northern Lights or Aurora spectral flow cytometers with Spectroflo software.
Software	FlowJo software v10.8.1
Cell population abundance	Purity was determined post-sort by analyzing an aliqout of the sorted material with a threshold of 95% purity accepted
Gating strategy	Immune cell populations were defined as: liver Kupffer cell (CD3-CD19-Ly6G-F4/80+TIMD4+ CD64+), liver pDC (CD19-B220+CD172+), liver neutrophil (F4/80-CD11b+Ly6G+), liver DC (CD11c+MHCII+), Liver B cell (CD19+B220+), spleen CD8 T-cell (CD3+CD4-CD8+) and spleen B cell (CD45+CD19+B220+). Bone marrow granulocyte were defined as CD45+CD11b+Ly6G+, bone marrow monocytes as CD45+CD11b+Ly6G-Ly6C+, liver monocytes as CD45+CD19-CD3e-TIMD4-Ly6C+, blood granulocytes as CD45+CD19-B220-CD11b+Ly6G+ and blood monocytes as CD45+CD19-B220-CD11b+Ly6G-Ly6Clo to hi. Cell populations were gated first on FSC-H and SSC-H to remove debris and FSC-H versus FSC-H to define singlets. Compensation (spectral unmixing) were always performed for each antibody that was used in the gating strategy and used to define positive and negative populations.

☒ Tick this box to confirm that a figure exemplifying the gating strategy is provided in the Supplementary Information.
