# Supplementary figures and images for: Kupffer cells dictate hepatic responses to the atherogenic dyslipidemic insult
Source: Nat Cardiovasc Res. 2024 Mar 11;3(3):356–71. doi: 10.1038/s44161-024-00448-6 (PMC11358021; doi:10.1038/s44161-024-00448-6)

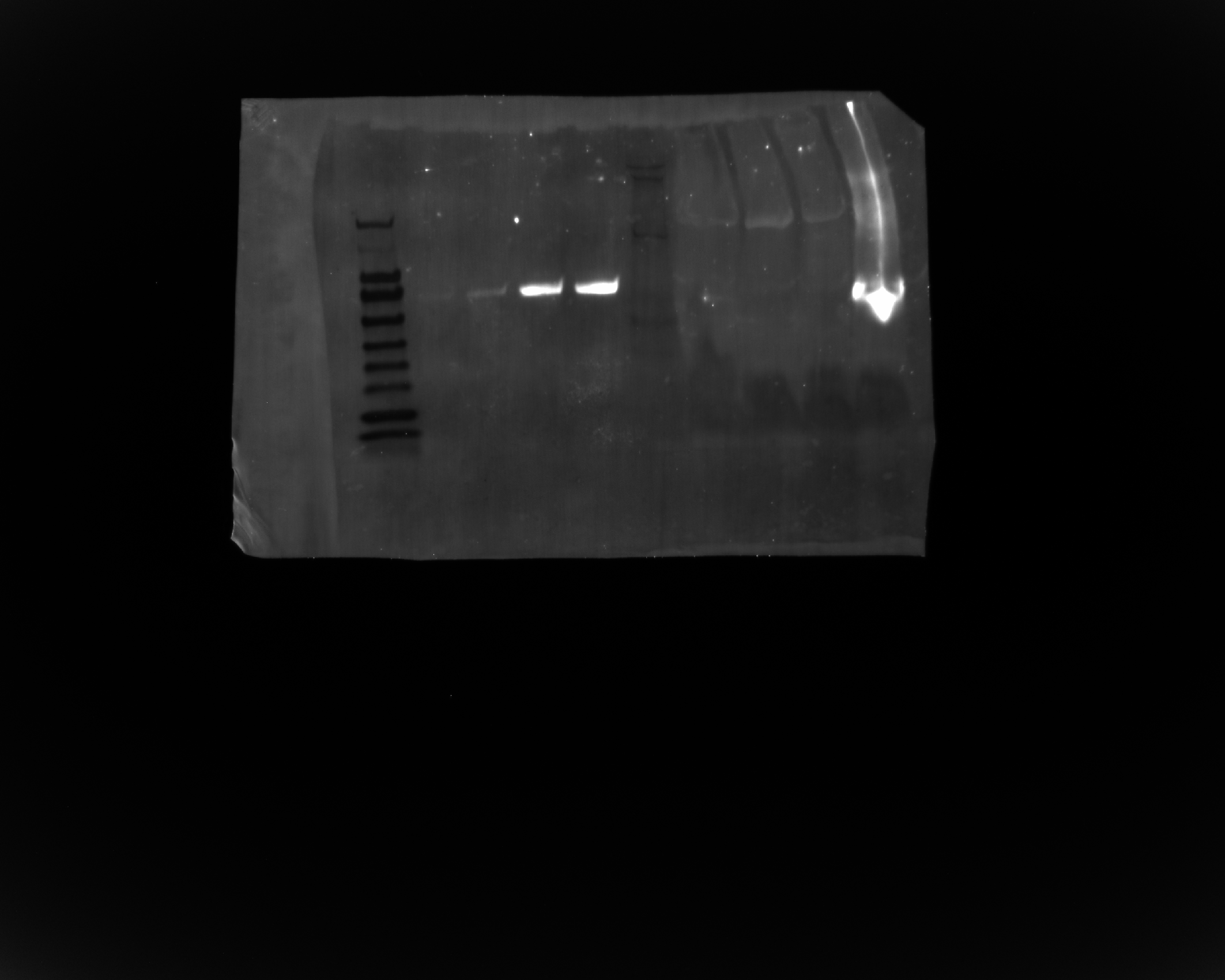

Supplement: Supplementary file 3 — Full western blot. [file 44161_2024_448_MOESM3_ESM.tif]
